# FHL1 mediates HOXA10 deacetylation via SIRT2 to enhance blastocyst-epithelial adhesion

**DOI:** 10.1038/s41420-022-01253-5

**Published:** 2022-11-22

**Authors:** Zhiwen Cao, Qiang Yan, Mei Zhang, Yingchun Zhu, Jingyu Liu, Yue Jiang, Xin Zhen, Manlin Xu, Qiuling Yue, Jidong Zhou, Quan Zhou, Xiaoying Wang, Lijun Ding, Haixiang Sun, Guijun Yan

**Affiliations:** 1 Center for Reproductive Medicine and Obstetrics and Gynecology, Nanjing Drum Tower Hospital, Nanjing University Medical School, Nanjing, China; 2 State Key Laboratory of Pharmaceutical Biotechnology, Nanjing University, Nanjing, China; 3 Reproductive medical center, Shanghai First Maternity, and Infant Hospital, Tongji University School of Medicine, No. 2699, West Gaoke Road, Pudong New District, 200120 Shanghai, China; 4 State Key Laboratory of Reproductive Medicine, Nanjing Medical University, Nanjing, China; 5 Center for Molecular Reproductive Medicine, Nanjing University, Nanjing, China

**Keywords:** Infertility, Biochemistry

## Abstract

Recurrent implantation failure (RIF) is a rather thorny problem in the clinical practice of assisted reproductive technology. Due to the complex aetiology of RIF, its pathogenesis is far from fully understood, and there is no effective treatment available. Here, We explored the regulatory mechanism of the four half-domains of LIM domain 1 (FHL1), which is significantly downregulated in the endometrium of RIF patients, in blastocyst-epithelial adhesion. Indeed, FHL1 expression was dramatically increased in normal female mid-secretory endometrial epithelial cells and was abnormally reduced in RIF patients. Furthermore, FHL1 overexpression promoted blastocyst-epithelial adhesion, and interfering with FHL1 expression in the mouse uterus significantly inhibited embryo implantation. Mechanistically, FHL1 did not regulate HOXA10 mRNA expression but increased HOXA10 protein stability and activated HOXA10, thereby promoting its regulation of downstream gene expression and the β3 integrin/FAK pathway. Meanwhile, FHL1 regulates HOXA10 function by increasing HOXA10 deacetylation through enhanced binding of HOXA10 and SIRT2. SIRT2-specific inhibitors can significantly inhibit this effect. In the endometrial epithelial cells of RIF patients, the correlation between FHL1 and HOXA10 and its downstream target genes has also been verified. Finally, our data indicated FHL1 is a regulatory molecule that promotes blastocyst-epithelial adhesion. Altogether, downstream dysfunction due to aberrant FHL1 expression is an important molecular basis for embryo implantation failure in patients with RIF and to provide new potential therapeutic targets.

## Introduction

Recurrent implantation failure (RIF) is defined as the failure of clinical pregnancy in a woman younger than 40 years following the transfer of at least 4 high-quality embryos in at least three fresh or frozen cycles [1, 2]. In recent years, RIF has become a most difficult clinical problem in assisted reproduction and is responsible for 8–10% of assisted reproductive technology failures [3]. In fact, the majority of these patients do not achieve a successful pregnancy despite the transfer of normally developing high-quality embryos. Impaired maternal endometrial receptivity is the key reason for the failure of embryo implantation in these patients [4–6]. Endometrial epithelial cells, which are under the control of oestrogen and progesterone, play a key role in the establishment of endometrial receptivity in the mid-secretory phase and mediate the first contact between the blastocyst and the maternal uterus [7]. Due to uncertainties and limited in-depth research on the pathophysiological mechanisms of impaired endometrial receptivity in RIF patients, there is still a lack of effective treatments for this intractable disease.

HOXA10 is periodically expressed in endometrial epithelial cells and stromal cells and is one of the most critical factors in the endometrium regulating embryo implantation [8–10]. Genetically engineered mouse studies have shown that normal embryos develop in HOXA10 knockout mice, but these mice show the specific deletion of cyclin-dependent kinase 4 and abnormal expression of cyclin D3 in utero, resulting in embryo implantation failure [11–14]. Furthermore, HOXA10 acts as a transcription factor in the endometrium to regulate stable adhesion during embryo implantation by activating key downstream target genes and the β3 integrin/FAK pathway [15–20]. In addition, HOXA10 has been reported to be associated with embryo implantation failure due to various endometrial diseases. IL-10 [21], m6A methylation [18], and microRNAs [22] all regulate endometrial receptivity in patients with endometriosis, adenomyosis, and RIF by affectting HOXA10 functionality.

The four half-domains of LIM domain 1 (FHL1) are semi-highly conserved LIM domains belonging to a family with five members: FHL1, FHL2, FHL3, FHL4, and FHL5 [23]. FHL4 is not expressed in human tissues, and FHL5 is specifically expressed in testis tissue [24]. They interact with certain protein kinases (ERK2, SPHK1, HDAC5, SIRT1, etc.) and transcription factors (SMAD4, P53, CREM, NFATC1, etc.) through the LIM domains of cysteine-rich double zinc finger structures to regulate cellular growth, differentiation, migration and cytoskeletal remodelling [24–26]. Based on sequencing data from the GEO database [27], we found that FHL1 levels were significantly reduced in endometrial tissue from RIF patients. This was confirmed by subsequent studies of FHL1 expression in numerous clinical endometrial tissue samples from RIF patients. Nevertheless, the role of FHL1 in the establishment of endometrial receptivity and RIF pathology in the uterus remains to be further explored. In this study, we revealed a novel mechanism by which abnormally expressed FHL1 in the endometrial epithelial cells of patients with RIF regulates embryo implantation, which may provide a new target for RIF therapy.

## Results

### FHL1 is cyclically expressed in the human endometrium but deficient in RIF patients

**Fig. 1:**
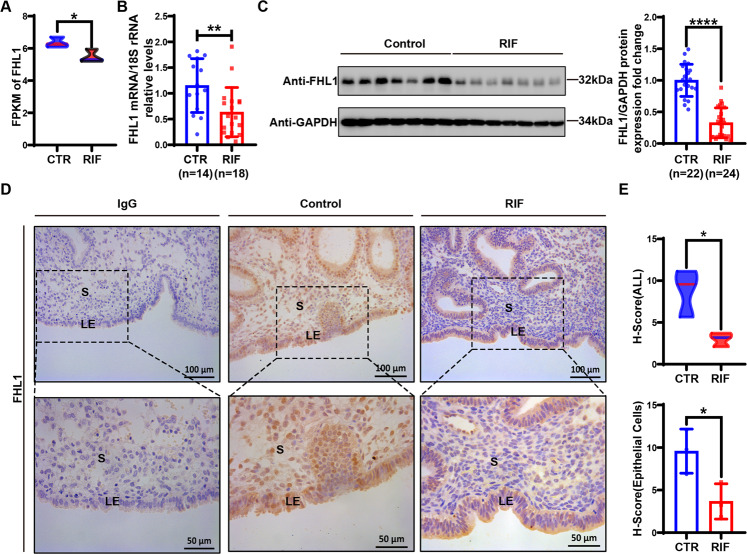
FHL1 expression is markedly decreased in endometrial cells of RIF patients. **A** GEO database data (GSE103465) show that FHL1 is expressed in the endometria of women with RIF and fertile controls. **B** The relative expression of FHL1 mRNA in RIF (*n* = 18) and CTR (fertile women, *n* = 14) human endometrial tissues was measured by qRT–PCR. The 18S gene was used as a loading control. **P < 0.01, by Student’s t test. **C** Western blot analysis to detect FHL1 in RIF (*n* = 24) and CTR (fertile woman, *n* = 22) human endometrial tissues. Normalization was performed with the GAPDH protein as a housekeeping protein. ***P < 0.001, by Student’s t test. **D**, **E** The localization of FHL1 expression in RIF (*n* = 3) and CTR (fertile woman, *n* = 3) human endometria was detected by immunohistochemistry. The total expression of FHL1 in RIF and CTR endometria and its expression in epithelial cells were analyzed. IgG was used as a negative control. GE: gland, S: stroma. *P < 0.05, by Student’s t test. Scale bar = 50 μm.

GEO database sequence data (GSE103465) suggested that FHL1 expression is reduced in the endometria of RIF patients (Fig. 1A). This was further examined by qRT–PCR and Western blot analyses of FHL1 expression in the endometrial tissues of RIF patients collected from our reproductive medicine centre (Fig. 1B, C). More importantly, we performed immunohistochemistry experiments and observed that FHL1 expression was particularly significantly reduced in endometrial epithelial cells from RIF patients compared with its expression in normal female endometrial tissue (Fig. 1D, E).

**Fig. 2:**
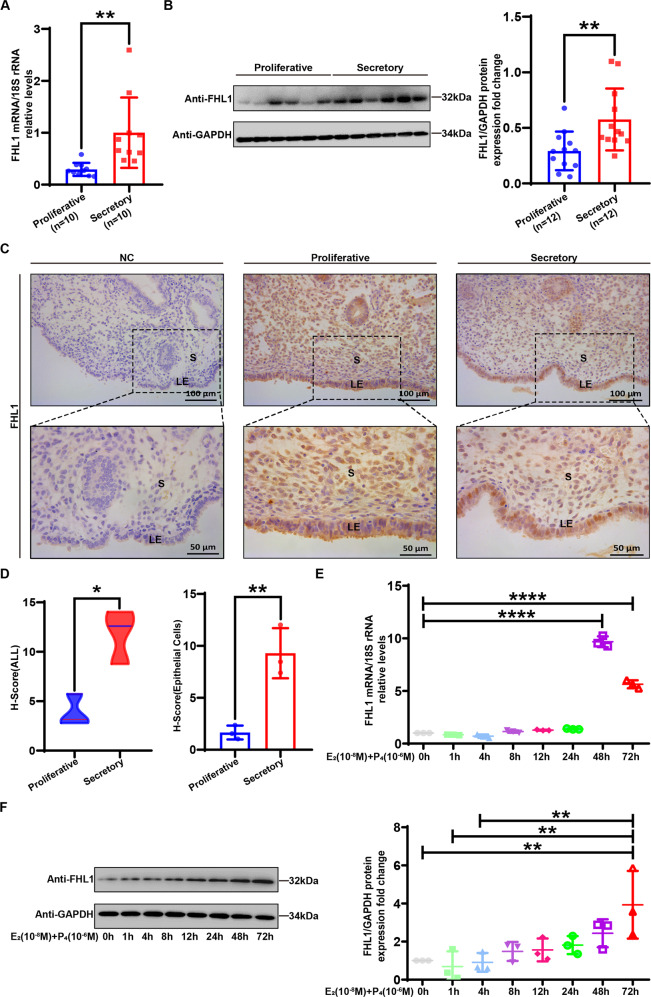
FHL1 is highly expressed in human mid-secretory-phase endometrial epithelial cells, regulated by oestrogen and progesterone. **A** The relative expression of FHL1 mRNA in secretory (*n* = 10) and proliferative (*n* = 10) human endometrial tissues was measured by qRT–PCR. The 18S gene was used as a loading control. **P < 0.01, by Student’s t test. **B** Western blot analysis to detect FHL1 in secretory (*n* = 12) and proliferative (*n* = 12) human endometrial tissues. Normalization was performed with the GAPDH protein as the housekeeping protein. **P < 0.01, by Student’s t test. **C**, **D** The localization of FHL1 expression in proliferative (*n* = 3) and secretory (*n* = 3) human endometria was detected by immunohistochemistry. The total expression of FHL1 in secretory and proliferative endometria and its expression in epithelial cells were analyzed. IgG was used as a negative control. GE: gland, S: stroma. *P < 0.05, **P < 0.01, by Student’s t test. Scale bar = 50 μm. **E**, **F** Ishikawa cells were stimulated with oestrogen (E2:10^−^^8^ M) combined with progesterone (P4:10^−6^ M) for 0–72 h, and the relative expression of FHL1 mRNA was measured by qRT–PCR. The 18S gene was used as a loading control. FHL1 protein expression was detected by Western blotting, and normalization was performed against GAPDH as a housekeeping protein. ***P < 0.001, ****P < 0.0001, by one-way ANOVA in (**E**, **F**).

To evaluate the expression and regulation of FHL1 in the endometrium during the human physiological cycle, we first investigated the expression of FHL1 and other family members (FHL2/FHL3) in the human endometrium. The analysis of the experimental results showed that the mRNA and protein expression levels of FHL1 in the endometrium were significantly increased in the mid-secretory phase (Fig. 2A, B). Relative to FHL1, mRNA expression of FHL2 and FHL3 accounted for a lower proportion of FHL family expression during the proliferative and secretory phases of the endometrium (Fig. S1A), and no significant differences were found (Fig. S1B, C). Furthermore, FHL1 was expressed in both human endometrial epithelial cells and stromal cells, as observed by immunohistochemical staining (Fig. 2C), and the changes in FHL1 expression were mainly concentrated in the epithelial cells of endometrial tissue in the mid-secretory phase rather than the proliferative phase (Fig. 2D). In addition, the experiments showed that the expression of FHL1 in endometrial epithelial cells increased in a time-dependent manner under combined stimulation by oestrogen and progesterone (Fig. 2E, F). Therefore, FHL1 is regulated by oestrogen and progesterone in endometrial epithelial cells and shows abnormal expression and localization changes in RIF.

### FHL1 is crucial for blastocyst-epithelial adhesion

**Fig. 3:**
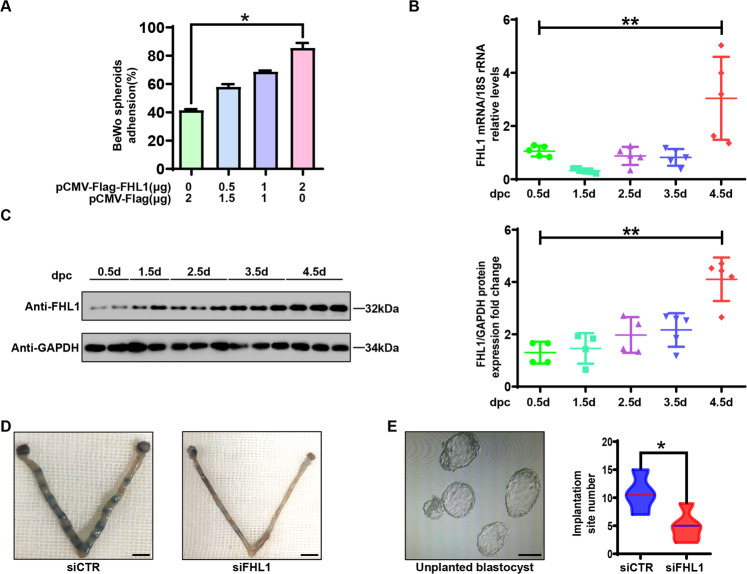
FHL1 is crucial for blastocyst-epithelial adhesion. **A** BeWo spheroids (150–200 μm in diameter, 50 per group) were attached to Flag-FHL1-transfected Ishikawa cells after 2 h of coculture. The percentage of BeWo ball adhesion efficiency was calculated. The average of three independent experiments was taken. *P < 0.05, by one-way ANOVA. **B** The relative expression of FHL1 mRNA in the peri-implantation phase of mice was measured by qRT–PCR. The 18S gene was used as a loading control. **P < 0.01, by one-way ANOVA. **C** Western blot analysis to detect FHL1 in the peri-implantation phase of mice. Normalization was performed against the GAPDH protein as a housekeeping protein. **P < 0.01, by one-way ANOVA. **D**, **E** FHL1 protein expression in mice was disrupted with siRNA against FHL1 (siFHL1) or a nonsilencing control (siNC). The expression of FHL1 in the siFHL1 group (*n* = 5) and siNC group (*n* = 4) was detected by Western blotting. Uteri from siFHL1 and control mice at 4.5 days of gestation were collected and stained with Chicago blue dye. The uterus was flushed, and unimplanted blastocysts were counted under a light microscope. *P < 0.05, by Student’s t test. Scale bar = 1 cm and 250 μm.

Because endometrial epithelial cells mediate the first contact between the blastocyst and the uterus, they are key to the establishment of endometrial receptivity and the adhesion of blastocysts [28]. Therefore, we further explored the function of FHL1 in endometrial epithelial cells. First, we constructed a widely used in vitro blastocyst-epithelial adhesion model from trophoblast BeWo spheres and Ishikawa cells [29–31]. The results presented in Fig. 3A showed that FHL1 overexpression in Ishikawa cells promoted blastocyst-epithelial adhesion in a concentration-dependent manner. A more than 40% increase in the blastocyst adhesion efficiency was observed in the FHL1 overexpression group relative to the control group (Fig. 3A). Furthermore, FHL1 expression increased in a time-dependent manner in the uteri of mice during implantation (Fig. 3B, C). Moreover, embryo implantation was significantly inhibited (10.75 ± 3.30 vs. 4.80 ± 2.68, *P* < 0.05) in the group in which FHL1 expression was knocked down in the uteri of mice (Fig. 3D, E). At the same time, the number of unplanted embryos increased significantly in the siFHL1 group, suggesting that interference with FHL1 expression inhibits blastocyst-epithelial adhesion.

### FHL1 is a newly identified HOXA10-interacting molecule

**Fig. 4:**
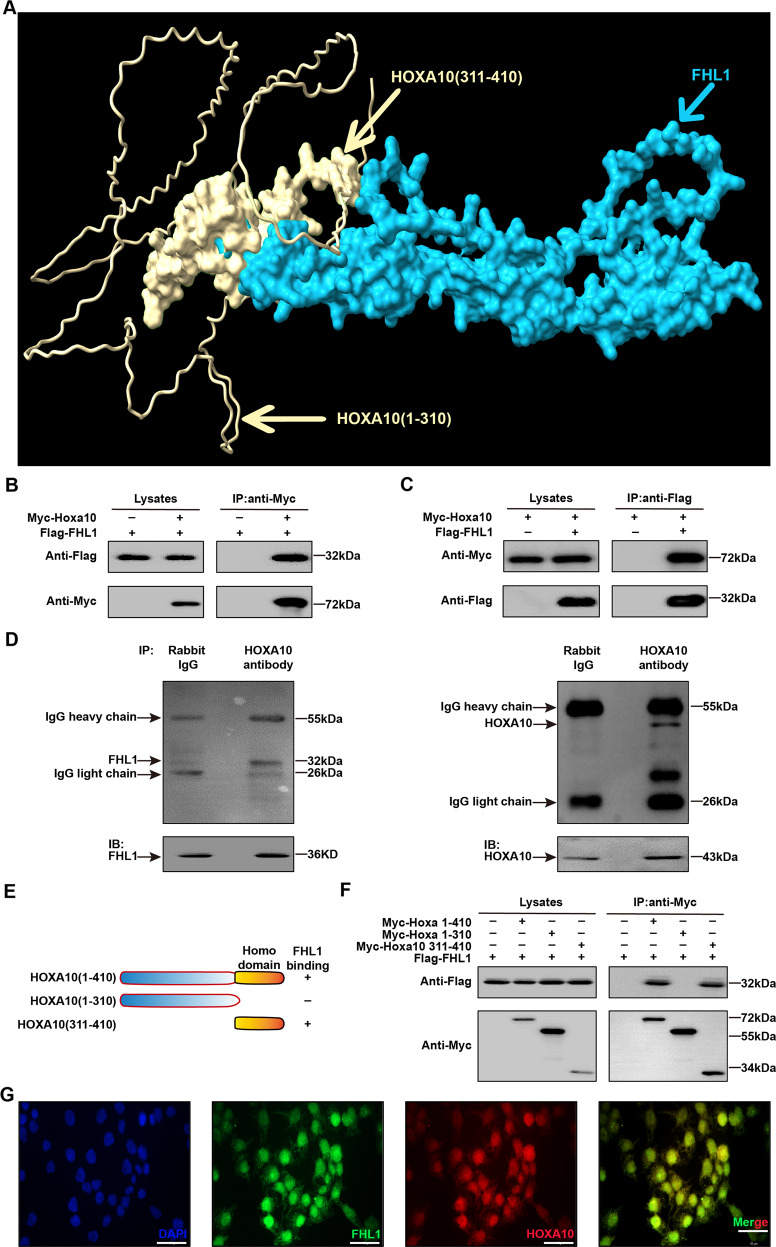
FHL1 is a newly identified HOXA10-interacting molecule. **A** Bioinformatics-based prediction of structural interactions of FHL1 with HOXA10 protein. **B**, **C** Anti-Myc/Flag immunoprecipitation followed by immunoblotting analysis of whole-cell lysates of HEK293T cells transfected with Flag-FHL1 either alone or together with Myc-HOXA10. **D** Ishikawa whole-cell lysates were subjected to anti-HOXA10 immunoprecipitation followed by immunoblotting analysis. Goat IgG was used as a negative control. **E** Schematic diagram of the HOXA10 mutants in Fig. 4F. **F** Flag-FHL1 and different Myc-HOXA10 mutants were cotransfected in HEK293T cells, and whole-cell lysates were collected for anti-Flag immunoprecipitation and immunoblotting analysis. **G** Endogenous FHL1 and HOXA10 significantly colocalized in Ishikawa nuclei.

We further sought to identify the key molecules through which FHL1 regulates the function of endometrial epithelial cells. Based on the amino acid sequence of the protein in the UniProt database, the FHL1 protein structure file was obtained from the Alphafold database and was analyzed by using Python and Chimrax software to screen for the possible FHL1-interacting protein HOXA10, a key regulator of endometrial receptivity [32]. Figure 4A shows a possible interaction between FHL1 and HOXA10 predicted by our own bioinformatics algorithm. Figure 4B, C shows that Myc-HOXA10 and Flag-FHL1 exhibited specific interactions in HEK293T cells, and the endogenous FHL1 and HOXA10 proteins also interacted in Ishikawa cells (Fig. 4D). Through domain analysis, we found that the C-terminus (311-410AA) of HOXA10, which contained the homeodomain, mediated its interaction with FHL1 (Fig. 4E, F). Furthermore, endogenous nuclear colocalization of FHL1 and HOXA10 was observed in Ishikawa cells by fluorescence microscopy (Fig. 4G). Therefore, we identified FHL1 as a novel interacting molecule of HOXA10.

### FHL1 acts as a HOXA10 coactivator of the β3 integrin/FAK pathway to promote blastocyst-epithelial adhesion

**Fig. 5:**
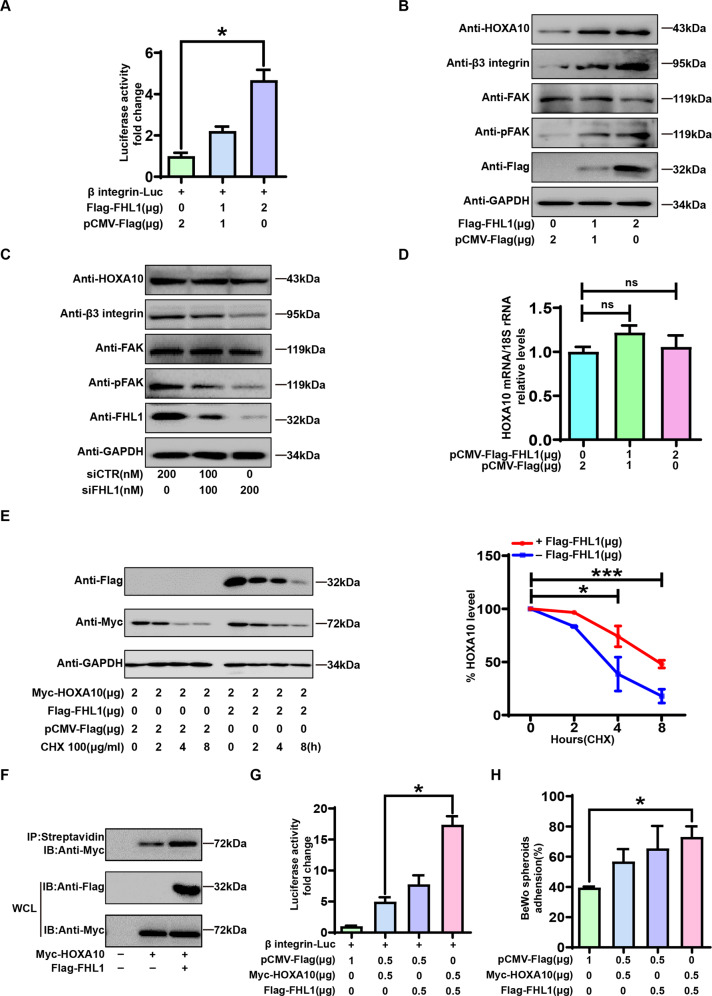
FHL1 acts as a HOXA10 coactivator of the β3 integrin/FAK pathway to promote blastocyst-epithelial adhesion. **A** Ishikawa cells were cotransfected with β integrin-Luc and Flag-FHL1 for 48 h. Luciferase activity was measured; *P < 0.05, by one-way ANOVA. **B** Flag-FHL1 (0–2 μg) was transfected into Ishikawa cells for 48 h. Western blotting was used to detect the expression of related proteins in the HOXA10/β3 integrin/FAK pathway. **C** siFHL1 (0–200 nM) was transfected into Ishikawa cells for 48 h. Western blotting was used to detect the expression of related proteins in the HOXA10/β3 integrin/FAK pathway. **D** Flag-FHL1 (0–2 μg) was transfected into Ishikawa cells for 48 h. qRT–PCR was used to detect the expression of HOXA10 mRNA. The 18S gene was used as a loading control. ns = no significant difference by Student’s t test. **E** Ishikawa cells were transfected with Flag-FHL1 (2 μg) and Myc-HOXA10 (2 μg) for 24 h. Cycloheximide (CHX, 10 μg/ml) was added to the cell culture medium, total protein was isolated, and FHL1 and HOXA10 protein levels were detected by immunoblotting at specific times. **F** Flag-FHL1 and Myc-HOXA10 were transfected into HEK293T cells, and the cell lysate was incubated with DNA probes and streptavidin Sepharose beads, and Western blot analysis was then performed on the extracts. **G** Ishikawa cells were cotransfected with β integrin-Luc, Flag-FHL1, and Myc-HOXA10 for 48 h. Luciferase activity was measured, *P < 0.05, by one-way ANOVA. **H** BeWo spheroids (150–200 μm in diameter, 50 per group) were attached to Flag-FHL1/Myc-HOXA10-transfected Ishikawa cells after 2 h of coculture. The percentage of BeWo ball adhesion efficiency was calculated. The average of three independent experiments was taken. *P < 0.05, by one-way ANOVA.

As shown in Fig. 5A–C, FHL1 overexpression promoted β3-integrin transcriptional activity downstream of HOXA10 and β3-integrin/FAK pathway activation. Unexpectedly, FHL1 did not affect HOXA10 mRNA expression levels (Fig. 5D). Further experiments showed that FHL1 increased the protein stability of HOXA10 after the cycloheximide stimulation of Ishikawa cells coexpressing FHL1 and HOXA10 for 0–8 h (Fig. 5E) and promoted the activation of HOXA10, thereby regulating the expression of downstream genes (Fig. 5F). On this basis, the luciferase reporter gene results showed that in Ishikawa cells with FHL1 and HOXA10 coexpression, HOXA10 increased the activity of the target gene β3 integrin more than 3-fold (Fig. 5G). More importantly, the coexpression of FHL1 with HOXA10 significantly enhanced the blastocyst-epithelial adhesion-promoting effect of HOXA10 (Fig. 5H). Collectively, these results suggest that FHL1 activates β3 integrin/FAK to promote blastocyst-epithelial adhesion by acting as a HOXA10 coactivator.

### FHL1 promotes the SIRT2-mediated deacetylation of HOXA10

**Fig. 6:**
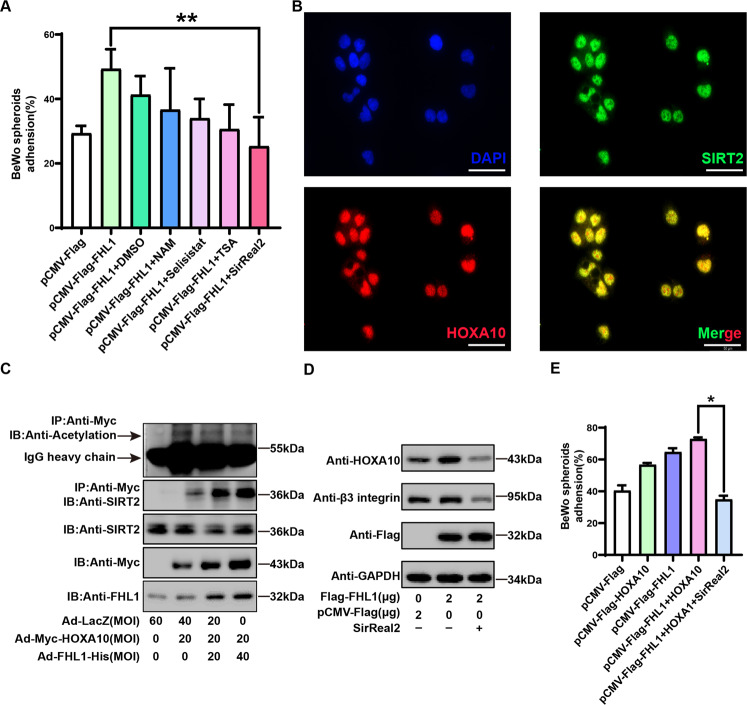
FHL1 promotes the SIRT2-mediated acetylation of HOXA10. **A** Ishikawa cells were transfected with Flag-FHL1, various deacetylation inhibitors were added, and BeWo spheroids (150–200 μm in diameter, 50 per group) were cocultured with Ishikawa cells for 2 h. The BeWo ball adhesion efficiency percentage was calculated. The average of three independent experiments was taken. Concentration of deacetylation inhibitor used in the experiment (Trichostatin A (TSA): 125 nM; Nicotinamide (NAM): 250 nM; Selisistat: 10 nM; SirReal2: 10 nM). *P < 0.05, one-way ANOVA. **B** Endogenous SIRT2 and HOXA10 significantly colocalized in Ishikawa nuclei. **C** Forty-eight hours after Ishikawa cells were transfected with Ad-LacZ (0–60 MOI), Ad-Myc-HOXA10 (0–20 MOI), and Ad-FHL1-His (0–40 MOI), whole-cell lysates were subjected to immunoblotting to detect SIRT2 and HOXA10 acetylation after anti-Myc immunoprecipitation. **D** Flag-FHL1 (0–2 μg) was transfected into Ishikawa cells for 48 h with the addition of SIRT2-specific inhibitor (SirReal2: 10 nM). Western blotting was used to detect the expression of the protein of interest. **E** Ishikawa cells were transfected with Flag-FHL1/Myc-HOXA10, a SIRT2-specific inhibitor (SirReal2: 10 nM) was added, and BeWo spheroids (150–200 μm in diameter, 50 per group) were cocultured with Ishikawa cells for 2 h. The BeWo ball adhesion efficiency percentage was calculated. The average of three independent experiments was taken. *P < 0.05, one-way ANOVA.

FHL family proteins often play a transcriptional regulatory role by recruiting deacetylases to transcription factor complexes and mediating transcription factor deacetylation [33, 34]. Therefore, we first screened a variety of potential deacetylation inhibitors of FHL1 in preliminary experiments, and we found that a SIRT2-specific inhibitor (SirReal2) significantly inhibited the FHL1-mediated promotion of endometrial blastocyst-epithelial adhesion (Fig. 6A). Furthermore, endogenous nuclear colocalization of SIRT2 and HOXA10 was observed in Ishikawa cells by fluorescence microscopy (Fig. 6B). Moreover, Fig. 6C shows that FHL1 overexpression in the endometrial epithelium enhanced the interaction of HOXA10 with SIRT2 and promoted HOXA10 deacetylation. More importantly, SirReal2 significantly inhibited the promotion effect of FHL1 on HOXA10 downstream target gene expression and blastocyst-epithelial adhesion (Fig. 6D, E). Thus, FHL1 regulates blastocyst-epithelial adhesion through the SIRT2-mediated deacetylation of HOXA10.

### FHL1 is correlated with HOXA10 and its downstream target gene β3 integrin in RIF

**Fig. 7:**
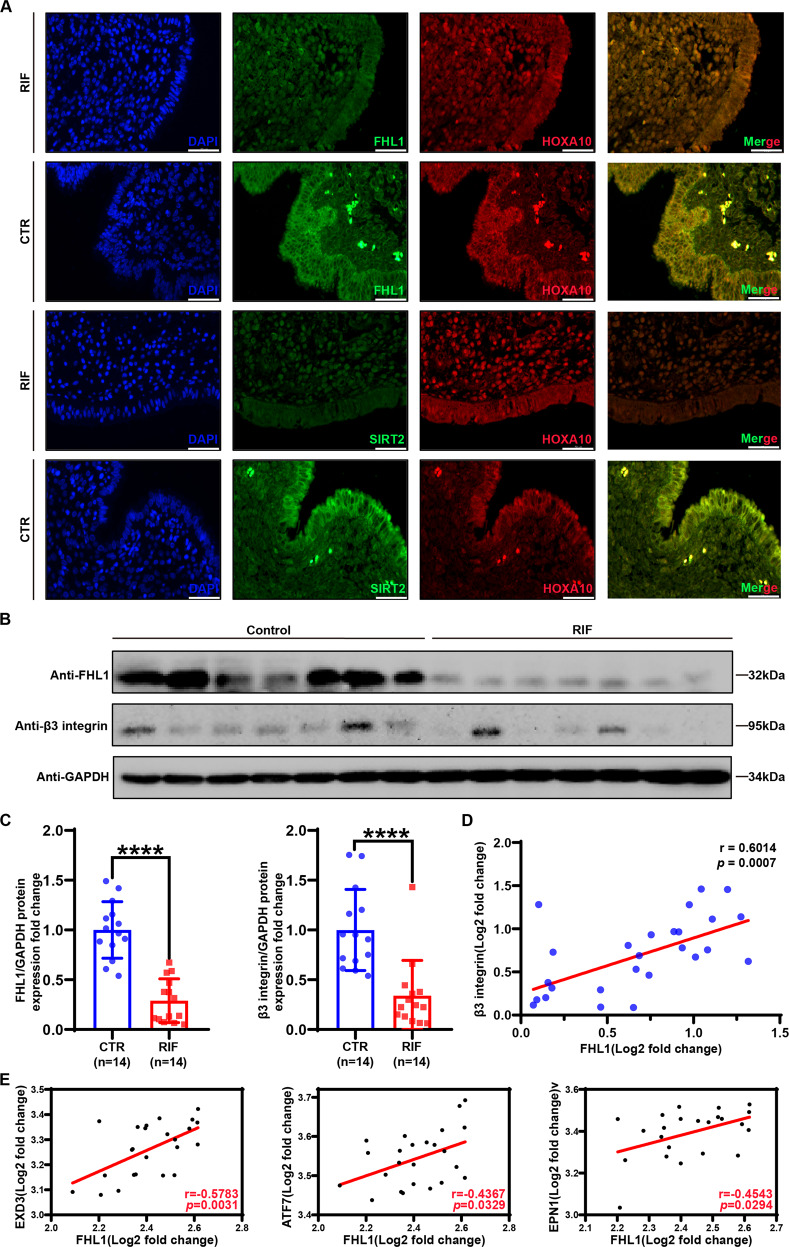
FHL1 interacts with HOXA10 and its expression is significantly reduced in RIF endometrial epithelial cells. **A** Immunohistofluorescence detection of FHL1/SIRT2 (green) and HOXA10 (red) in endometrial tissue from RIF and control groups. Scale bar = 10 μm. **B**, **C** Western blot analysis to detect FHL1 and β3 integrin in RIF (*n* = 14) and CTR (fertile woman, *n* = 14) human endometrial tissues. Normalization was performed with the GAPDH protein as a housekeeping protein. ***P < 0.001, by Student’s t test. **D** Correlation test for quantitative protein levels in human endometrial samples. The Pearson correlation test analyses the *r* and *P* values of protein correlations. **E** Correlation scores between FHL1 and mRNA levels of HOXA10 downstream target genes in RIF patients endometrial tissue in the midsecretory stage (*n* = 24). The Pearson correlation test analyses the *r* and *P* values of protein correlations. The data are derived from the GSE111974 dataset.

We next examined the correlation between FHL1 and HOXA10 function in the endometria of RIF patients. The colocalization of FHL1, SIRT2, and HOXA10 was significantly decreased in the endometrial epithelial cells of RIF patients (Fig. 7A). Moreover, Western blotting verified the expression of FHL1 and β3 integrin was reduced in RIF patients (Fig. 7B, C) and correlation analysis showed that FHL1 was significantly correlated with β3 integrin (*r* = 0.6014, *p* = 0.0007) (Fig. 7D). Similarly, the sequencing data (GSE111974) [35] of RIF patients showed that FHL1 was significantly correlated with other downstream target genes of HOXA10, such as EXD3 (*r* = 0.5783, *p* = 0.0031), ATF7 (*r* = 0.4367, *p* = 0.0329), EPN1 (*r* = 0.4543, *p* = 0.0294) (Fig. 7D). These results suggest a consistent role of FHL1 in regulating HOXA10 function in RIF.

## Discussion

**Fig. 8:**
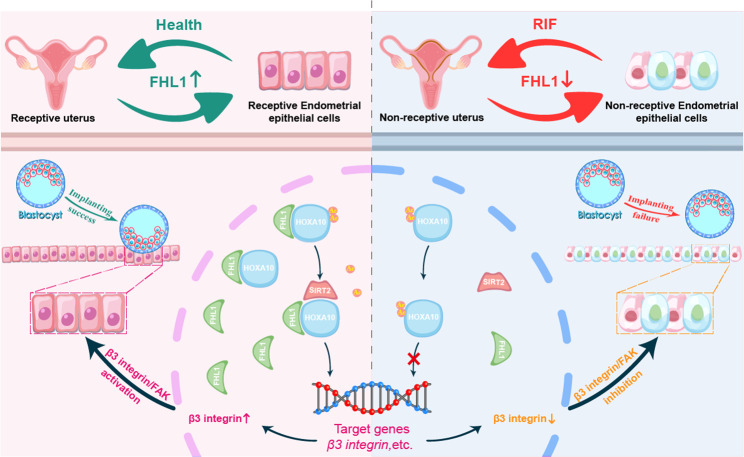
Schematic diagram of the role of FHL1-mediated HOXA10 deacetylation in the regulation of blastocyst epithelial adhesion in RIF patients and fertile controls. In endometrial epithelial cells, FHL1 increases HOXA10 deacetylation by promoting the interaction of SIRT2 and HOXA10, thereby enhancing HOXA10 target gene transcription and downstream pathway activation. This role of FHL1 in the endometrium involves the establishment of endometrial receptivity and the failure of blastocyst epithelial adhesion in RIF patients.

In conclusion, this study revealed the role of FHL1 in regulating endometrial receptivity and blastocyst-epithelial adhesion. This newly identified mechanism is independent of the regulation of HOXA10 mRNA expression but plays a transcriptional regulatory role by regulating HOXA10 deacetylation (Fig. 8). This role of FHL1 in the endometrium is involved in the establishment of endometrial receptivity for embryo implantation in RIF patients.

Endometrial epithelial cells are a very special cell type that plays a key role in switching between endometrial receptivity and nonreceptivity [36, 37]. In the absence of endometrial epithelial cells, embryos in the implanted state can undergo implantation without hormonal regulation [38, 39]. Embryo implantation is only permitted by the transformation of cyclic endometrial epithelial cells, regulated by oestrogen and progesterone, into a receptive endometrium in the mid-secretory phase [40]. In endometrial epithelial cells, FHL1 is regulated by oestrogen and progesterone, and its expression is increased in the mid-secretory phase, whereas abnormally reduced FHL1 expression is observed in RIF patients. Therefore, we first investigated the function of FHL1 in endometrial epithelial cells, although we also observed that FHL1 was relatively weakly expressed in endometrial stromal cells. Whether FHL1 is involved in the functional regulation of endometrial stromal cells still needs to be explored further.

As a member of the FHL family, FHL1 consists of four half-LIM domains. The LIM domain is a stable tertiary structure containing a Zn^2+^ binding pocket that can mediate the interactions between proteins such as transcription factors, protein kinases, and structural proteins [24]. FHL family members cannot directly bind DNA and instead act as coactivators or corepressors of transcription factors by binding these proteins [41, 42]. Previous studies have shown that FHL1 binds the oestrogen receptor transcription factor to regulate breast cancer cell growth and that the binding of FHL2 to the transcription factor E4F1 inhibits its transcriptional regulation and promotes cell proliferation [41, 43]. We also found that FHL1 can bind to the transcription factor HOXA10 and regulate its downstream gene expression to promote endometrial receptivity and blastocyst epithelial adhesion.

Recent studies have shown that inhibitors of histone deacetylases (HDACs), TSA, and the deacetylase SIRT1 affect blastocyst-epithelial cell adhesion and invasion through protein acetylation mechanisms, but their specific target molecules have not been identified [44]. This study found that in endometrial epithelial cells, FHL1 overexpression can promote blastocyst epithelial adhesion, and this promotion can be inhibited by a specific inhibitor of SIRT2, a member of the sirtuin family. Previous studies have shown that in addition to directly binding and regulating transcription factor function, the FHL1 family can also participate in the regulation of transcription factor function by interacting with various acetylases and deacetylases. For example, FHL1 regulates gene transcription driven by the transcription factor MEF2 in rat cardiomyocytes by promoting the phosphorylation of the HDAC5 protein [45]. The interaction of FHL2 and CBP/p300 increases the acetylation of β-catenin and synergistically regulates the expression of androgen receptor-responsive genes [46]. More interestingly, FHL2 can promote the interaction of SIRT1 with FOXO1, increase the SIRT1-mediated deacetylation of FOXO1, and thus, inhibit the transcriptional activity of FOXO1 [33]. Our study showed that FHL1 can promote the mutual binding of SIRT2 and HOXA10, increase the SIRT2-mediated deacetylation of HOXA10, and thus promote the transcriptional activity of HOXA10. Therefore, it is speculated that FHL1 and even other FHL family members form a “socket”-like protein structure based on their multiple interconnected LIM domains similar to the pocket structure, allowing these proteins to mediate the interactions between proteins and, thus, play a role in transcriptional regulation. Furthermore, whether FHL1 regulates blastocyst epithelial adhesion by mediating the interaction of other deacetylases with HOXA10 or with other transcription factors remains to be further investigated.

Because of the critical role of FHL1 in embryo implantation, we also identified proteins that interact with FHL1, with a particular focus on HOXA10. Multiple studies have shown that HOXA10 dysfunction is associated with impaired endometrial receptivity and RIF outcomes [47, 48]. However, HOXA10 function is regulated by DNA methylation [49], circular RNAs [50], lncRNAs [51], and various posttranslational modifications of proteins [17, 52]. In recent years, the important roles of various posttranslational modifications of the HOXA10 protein in the regulation of endometrial receptivity have been elucidated. These modifications include protein sumoylation [52], Ca^+^-dependent proteolysis [15], protein acetylation [17], and protein ubiquitination [53], all of which have been found to regulate embryo implantation when they occur on HOXA10. Our previous study showed that p300/CREB-binding protein-associated factor (PCAF) inhibited embryo implantation by increasing HOXA10 acetylation, thereby downregulating HOXA10 protein stability and inhibiting HOXA10 transcriptional activity [17]. In this study, we found that FHL1 promoted embryo implantation by increasing HOXA10 deacetylation, upregulating HOXA10 protein stability, and enhancing HOXA10 transcriptional activity. This contributed to revealing the yin and yang of acetylation and deacetylation in the regulation of embryo implantation mediated by HOXA10. Furthermore, although HOXA10 is regulated by multiple modifications, the consequences of these modifications all affect HOXA10 protein stability. It is well established that extensive regulation and constraints exist among different protein posttranslational modifications. For example, acetylation can compete with ubiquitination for the same lysine site to inhibit proteasomal degradation [54], while it can accelerate protein degradation by recruiting ubiquitin ligases [55]. This suggests that HOXA10 function may be regulated by a protein modification network. Therefore, revealing the relationships of this protein modification network will provide multiple specific therapeutic targets for the clinical treatment of RIF patients.

In conclusion, this study showed that FHL1 promotes the deacetylation of HOXA10 by promoting the binding of SIRT2 to HOXA10, thereby enhancing the activation of downstream target genes and pathways by HOXA10, which is a new mechanism regulating endometrial receptivity and blastocyst-epithelial adhesion. This work also provides new insights into potential therapeutic targets for RIF therapy.

## Materials and methods

### Ethics approval

This study was conducted in accordance with the recommendations of the Drum Tower Hospital Ethics Committee Clinical Trials Guidelines. Informed consent was obtained from participants for the collection of clinical samples associated with the experiment. This research is carried out in accordance with the approval of Construction and Management of the Nanjing Multic-centre Biobank Project (No. 2013-081-01, Registered Dec 10, 2013).

### Patients

From June 2019 to December 2021, 46 women (aged 26–39 years old) who were treated in the reproductive medicine of Drum Tower Hospital participated in the study. All women exhibited normal menstrual cycles, and none had received hormone therapy in the past three months. The exclusion criteria included polycystic ovary syndrome (PCOS), endometriosis and other ovarian and endometrial diseases. The inclusion and exclusion criteria for RIF endometrial tissue were as follows: samples must come from women younger than 40 years of age with unsuccessful pregnancy following the transfer of at least 4 high-quality split embryos or 2 blastocysts in at least three fresh or frozen cycles [2]; samples from women with adenomyosis, endometriosis, an endometrial thickness less than 7 cm, intrauterine adhesions, uterine malformation or other factors were excluded; the chromosome analysis results of both sides were normal and samples from women with recurrent miscarriage were excluded. Twenty-four women with RIF participated in this study, and the control group consisted of 22 women with male-factor infertility who achieved successful clinical pregnancies in 1 or 2 subsequent IVF cycles. Patient details are listed in Table S1.

### Western blot analysis

Endometrial tissues or cells were collected and subjected to protein extraction, and the protein concentration was determined. Protein lysates (18–20 μg) were separated by SDS–PAGE, and PVDF membranes were used for protein transfer. The membranes were blocked with 5% milk at room temperature for 1 h. They were then incubated with anti-FHL1 (Abcam, 1:1000, ab49241), anti-HOXA10 (Santa Cruz, 1:1000, sc-271953), anti-SIRT2 (Abcam, 1:1000, ab211033), anti-FAK (Abways, 1:1000, 12363-1-AP), anti-pFAK (Bioworld, 1:1000, 103571-AP), anti-β3 Integrin (Bioworld, 1:500, BS3660), anti-Flag (Sigma, 1:10,000, A8592), anti-Myc (Invitrogen, 1:10,000, 46-0709), and anti-GAPDH (Bioworld, 1:10,000, AP0063) primary antibodies overnight at 4 °C. Specific secondary antibodies were then added, and the membrane was further incubated for 1 h at room temperature. Proteins were then detected using an enhanced chemiluminescence kit (ClarityTM Western ECL Substrate, Bio-Rad). The ratio of the corresponding GAPDH grey value was analyzed by ImageJ software, and the value was normalized as the relative protein content.

### RNA isolation and qRT–PCR

After the collection of cells and endometrial tissue, total RNA was isolated and extracted by adding TRIzol (Thermo Fisher Scientific, USA) reagent according to the kit instructions. The RNA was subsequently reverse transcribed into cDNA following the instructions of a qRT–PCR kit (ABI Company, Oyster Bay, NY, USA). For qRT-qPCR analysis, SYBR Green Master Mix (Takara) and an Applied Biosystems 7500 Real-time PCR System (USA) were used to quantify expression. GAPDH or 18S rRNA was used as an internal control for FHL1, FHL2, FHL3, and HOXA10. Table S2 contains the primer sequences related to the experiment. The assay data were used to calculate relative mRNA expression changes using the 2-ΔΔCt method. Each sample was analyzed three times independently, and three replicate wells were set up for all samples.

### Immunohistochemistry (IHC)

After the dehydration and embedding of the tissue and dewaxing of 5 μm slices, antigen repair was performed using citrate buffer solution. At room temperature, 3% hydrogen peroxide was used to block endogenous peroxidase. Then, the cells were blocked with 5% goat serum for 45 min. Incubation with an anti-FHL1 primary antibody (1:200; Abcam, ab49241) was then performed overnight at 4 °C. The sections were subsequently incubated with a goat anti-rabbit secondary antibody for 30 min at 37 °C. Finally, the sections were stained with 3,3′-diaminobenzidine (DAB). The negative control was rabbit IgG. Digital images were acquired with a Leica DM 2000 microscope (Lecia, Wetzlar, Germany). Semiquantitative analysis was performed using ImageJ software.

### Immunofluorescence staining

First, 4% paraformaldehyde (Beyotime) was used to fix Ishikawa cells for 20 min. Subsequently, 0.1% Triton X-100 in PBS was used to permeabilize the cells for 10 min. Next, 3% BSA was used to block nonspecific sites for 1 h at 37 °C. The cells were then incubated overnight at 4 °C with primary antibodies against SIRT2 (1:200; Abcam, ab211033), HOXA10 (1:50, Santa Cruz, sc-271953) and FHL1 (1:100; Abcam, USA, ab255828). This was followed by further incubation with a fluorochrome-conjugated secondary antibody (Invitrogen) for 1 h at room temperature. Nuclei were stained using DAPI (Sigma–Aldrich) for 10 min. A fluorescence microscope (Olympus, FV10i) was used for photo acquisition.

### Transfection and luciferase assays

Ishikawa cells at a 75–80% density in 24-well plates were then transfected with β3 integrin-Luc (a luciferase reporter plasmid constructed from a plasmid reported in a previous study [16]) and Renilla luciferase and expression plasmids for 48 h. The Dual-Luciferase Assay System (Promega, USA) was used to analyze cell lysates for luciferase activity. Measurements were subsequently performed using a Centro XS3 LB 960 luminescence counter (Berthold Technologies, Germany) according to the manufacturer’s instructions. The activity of cotransfected Renilla luciferase was used for normalization.

### Assay of BeWo spheroid attachment to Ishikawa cells

We used widely employed in vitro adhesion models constructed from human choriocarcinoma BeWo and Ishikawa cells [29–31]. After reaching 80% confluence, BeWo cells were harvested using 0.25% trypsin (Gibco, USA). A single-cell suspension of BeWo cell suspensions was then transferred to a 35 mm^2^ dish coated with polyHEMA (Sigma) to form small spheroids that were 150–200 μm in diameter. The spheroids were then added to a monolayer of Ishikawa cells. PBS (GenMed Scientifics) containing Ca^2+^ (0.1 mg/L) and Mg^2+^ (0.1 mg/L) was used to wash away nonadherent spheroids after 2 h of spheroid adhesion. The adhesion rate is the number of adherent spheres divided by the percentage of the total number of spheres added to Ishikawa cells.

### Coimmunoprecipitation

Flag-FHL1 and Myc-HOXA10 plasmids were transiently cotransfected into HEK293T cells. After 48 h of plasmid transfection, the cells were harvested by washing twice with precooled 4 °C PBS. The whole-cell lysate containing protease inhibitors was added, followed by sonication and transfer to a 4 °C shaker for 30 min. Protein A/G PLUS-Sepharose beads (Abmart, China) were incubated with the same mass of total protein (500 μg) on a shaker at 4 °C for 2 h. After brief centrifugation, the supernatant was bound to 30 μl of Myc antibody-conjugated beads (Invitrogen) or 30 μl Flag antibody-conjugated beads (Sigma–Aldrich) overnight at 4 °C on a shaker. Further analysis was performed by Western blotting using an anti-Flag M2 antibody (Sigma–Aldrich) and an anti-Myc antibody (Thermo Scientific, USA).

For the immunoprecipitation assay of FHL1 with HOXA10 in Ishikawa cells, 1 mg of a total protein lysate was added to Protein A/G PLUS-Sepharose beads, and the mixture was incubated for 2 h at 4 °C on a shaker. After centrifugation at 8000 × *g* for 1.5 min at 4 °C, the supernatant was collected and incubated with an FHL1 antibody, HOXA10 antibody, and goat IgG antibody overnight at 4 °C on a shaker. The products were then bound to Protein A/G PLUS-Sepharose beads for 2 h at 4 °C and washed 3 times. Subsequently, they were used for Western blot detection.

### Mouse experiments

All mice used in this experiment were approved by the Animal Ethics Committee of Nanjing Drum Tower Hospital (SYXK 2019-0059). ICR mice were purchased from the Institute of Model Animals, Nanjing University (Nanjing, China). Mice were housed in SPF animal rooms and maintained at a constant temperature of 22–26 °C and relative humidity of 50–60% under day and night cycles of 12 h each. Pregnancy following the mating of 8-week-old female and fertile male mice was verified on the day of vaginal plug appearance (recorded as 0.5 dpc). The uteri of 0.5–4.5 dpc mice were collected and stored in liquid nitrogen. The expression of FHL1 in the peri-implantation uterus was detected by immunoblotting. In Fig. 3E, 20 µl (200 nmol) siFHL1 and siCTR were injected into the uterine horns of mice at 1.5 dpc. After the intravenous injection of Chicago blue in 4.5 dpc mice, the mouse uteri were collected to observe embryo implantation. The uterine cavity was rinsed with normal saline to observe whether any unimplanted embryos remained.

### Statistical analysis

We used SPSS 18 software (IBM Corporation, USA) to analyze the collected data, and measurement data are shown as the mean ± SEM. One-way analysis of variance was used for the statistical analysis of multiple groups of data. After the statistical analysis of the experimental data, calculated *P* values of less than 0.05 were considered statistically significant.

## Supplementary information

Fig S1. FHL1, FHL2, and FHL3 are expressed in the proliferative and secretory stages of the endometrium.

Table S1 Clinical characteristics of the women involved in this research

Table S2 Primers used in this research

Original Data File

## Data Availability

All data for this study are available from the respective authors upon reasonable request.
