# Supplementary material for: FHL1 mediates HOXA10 deacetylation via SIRT2 to enhance blastocyst-epithelial adhesion
Source: Cell Death Discov. 2022 Nov 22;8:461. doi: 10.1038/s41420-022-01253-5 (PMC9684570; doi:10.1038/s41420-022-01253-5)
Supplement: Supplementary file 1 — Fig S1. FHL1, FHL2, and FHL3 are expressed in the proliferative and secretory stages of the endometrium. [file 41420_2022_1253_MOESM1_ESM.pdf]

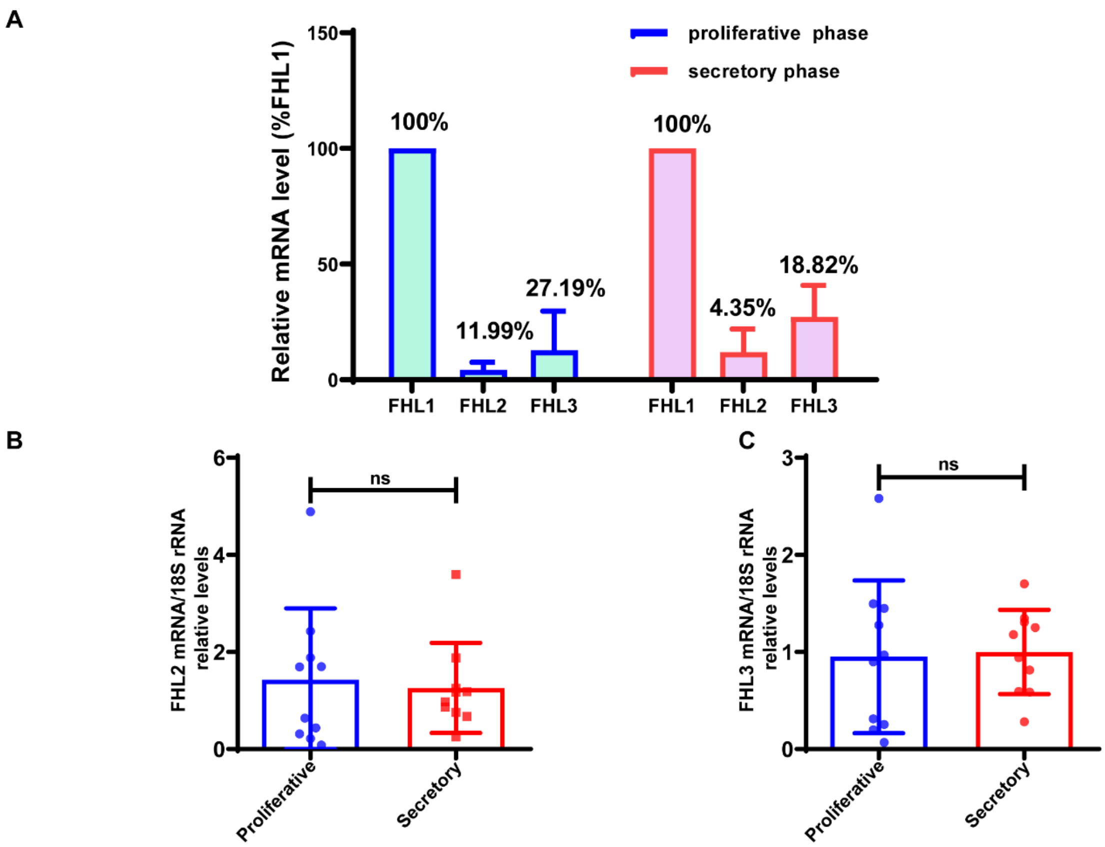

Fig S1. **FHL1, FHL2, and FHL3 are expressed in the proliferative and secretory stages of the endometrium.**

A) The relative mRNA expression ratios of FHL1, FHL2, and FHL3 during endometrial proliferation and secretion.

B-C) The relative mRNA expression levels of FHL2 and FHL3 during endometrial proliferation and secretion. The 18S gene was used as a loading control. ns=no significant difference by Student's t test.
