# Supplementary material for: FHL1 mediates HOXA10 deacetylation via SIRT2 to enhance blastocyst-epithelial adhesion
Source: Cell Death Discov. 2022 Nov 22;8:461. doi: 10.1038/s41420-022-01253-5 (PMC9684570; doi:10.1038/s41420-022-01253-5)
Supplement: Supplementary file 2 — Table S1 Clinical characteristics of the women involved in this research [file 41420_2022_1253_MOESM2_ESM.docx]

**Table S1 Clinical characteristics of the women involved in this reserach**

| Variables | Control groups  ( n = 22) | RIF patients  ( n = 24) | p value |
| --- | --- | --- | --- |
| Age (y) | 30.680±2.079 | 32.000±3.683 | 0.088 |
| BMI (kg/m^2^) | 22.24±4.167 | 21.800±2.349 | 0.672 |
| FSH (mIU/ml) | 8.095±1.876 | 8.194±2.031 | 0.864 |
| LH (mIU/ml) | 5.667±2.855 | 4.914±3.495 | 0.096 |
| Estradiol (pg/ml) | 48.200±30.680 | 44.850±19.540 | 0.664 |
| PRL (ng/ml) | 15.230±7.626 | 14.08±5.205 | 0.549 |
| T (ng/ml) | 0.4709±0.393 | 0.4096±0.393 | 0.537 |
| Number of embryo per transfer, n% | 1.636±0.492 | 1.594±0.310 | 0.729 |

Measurement data were expressed as mean ± SD, and normally distributed data were analyzed by unpaired t test.
