# Supplementary material for: FHL1 mediates HOXA10 deacetylation via SIRT2 to enhance blastocyst-epithelial adhesion
Source: Cell Death Discov. 2022 Nov 22;8:461. doi: 10.1038/s41420-022-01253-5 (PMC9684570; doi:10.1038/s41420-022-01253-5)
Supplement: Supplementary file 3 — Table S2 Primers used in this research [file 41420_2022_1253_MOESM3_ESM.docx]

**Table S2 Primers used in this reserach**

| Primer | Forward Sequence(5’→3’) | Reverse Sequence(5’→3’) | |
| --- | --- | --- | --- |
| Human |  | |  |
| FHL1 | TGCTGCCTGAAATGCTTTGAC | | GCCAGAAGCGGTTCTTATAGTG |
| FHL2 | AGAGTTTCATCCCCAAAGACAA | | AGTTCAGGCAGTAGGCAAAGTC |
| FHL3 | GGAGTGACATACCGTGATC | | GCAGGAGAAGCAGTTGTG |
| HOXA10 | AGGTGGACGCTGCGGCTAATCTCTA | | GCCCCTTCCGAGAGCAGCAAAG |
| 18s | CGGCTACCACATCCAAGGAA | | CTGGAATTACCGCGGCT |
| Mouse |  | |  |
| FHL1 | CTGTGGAATGTCATTTCTGTGC | | AGTTCCCAAAGTACATAAGAGGAG |
| 18s | ATGGCCGTTCTTAGTTGGTG | | CGGACATCTAAGGGCATCAC |
