# Supplementary material for: FHL1 mediates HOXA10 deacetylation via SIRT2 to enhance blastocyst-epithelial adhesion
Source: Cell Death Discov. 2022 Nov 22;8:461. doi: 10.1038/s41420-022-01253-5 (PMC9684570; doi:10.1038/s41420-022-01253-5)

Figure 1C　original picture (CTR VS RIF n=22 vs 24)

Endometrium FER(n=7) vs RIF(n=7)

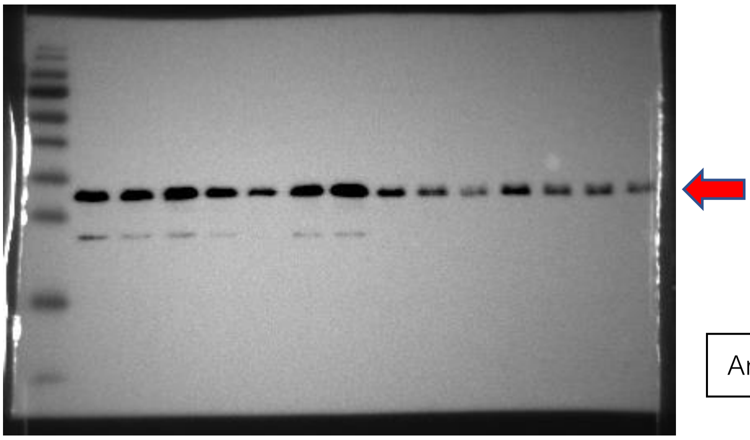

Anti-FHL1

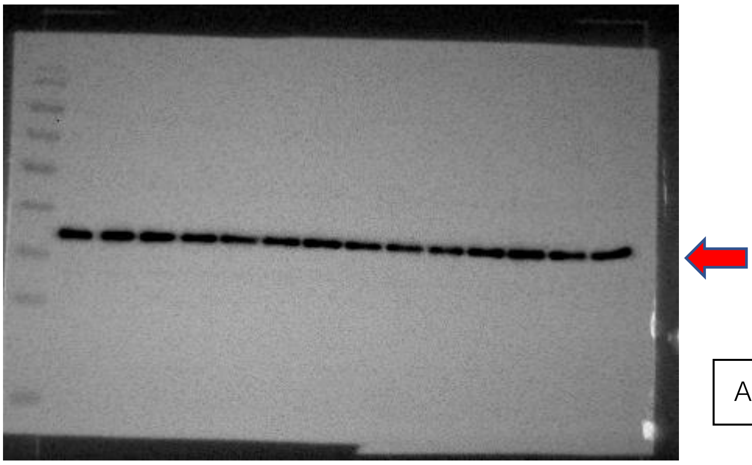

Anti-GAPDH

Endometrium CTR(n=8)

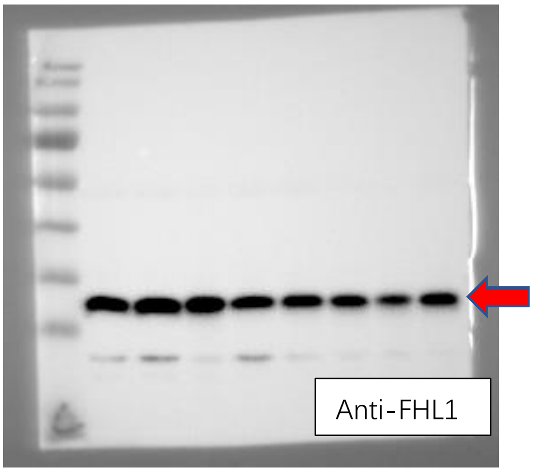

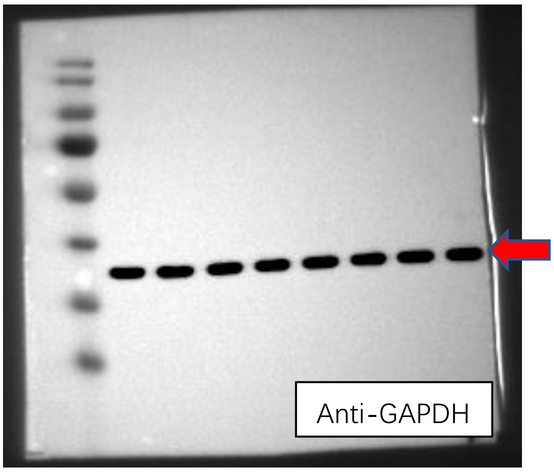

Endometrium RIF(n=10)

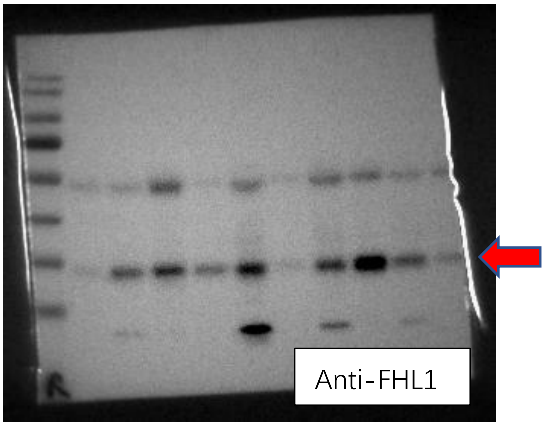

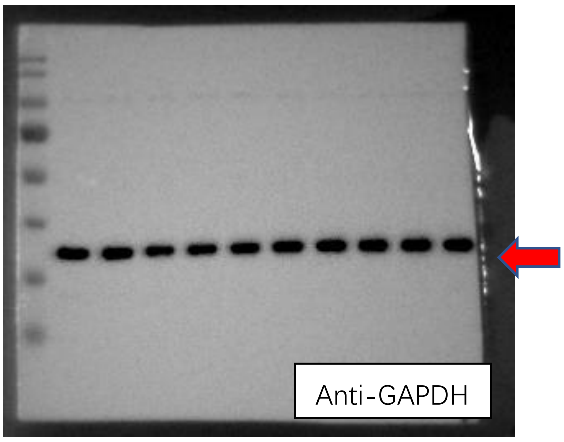

Endometrium FER(n=7) vs RIF(n=7)

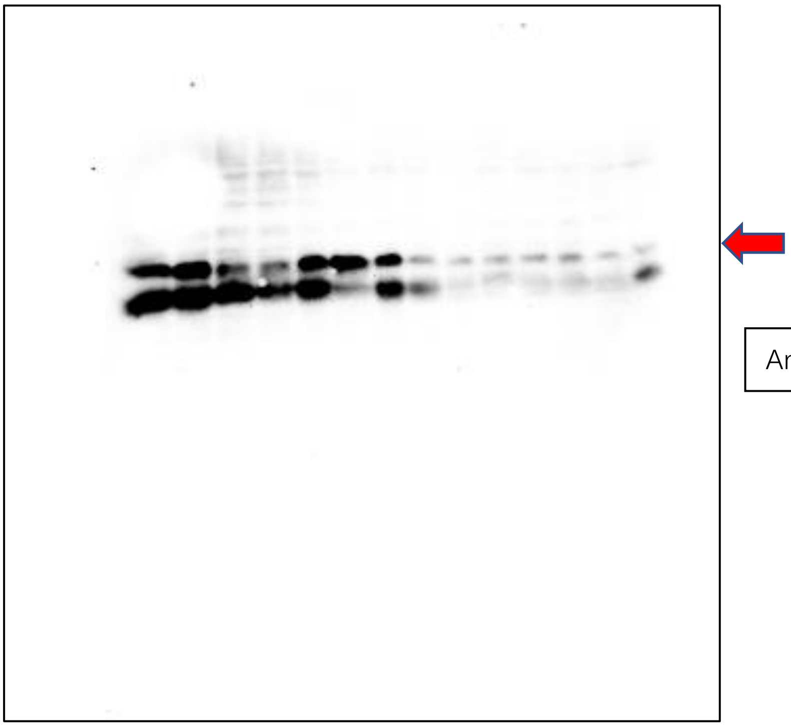

Anti-FHL1

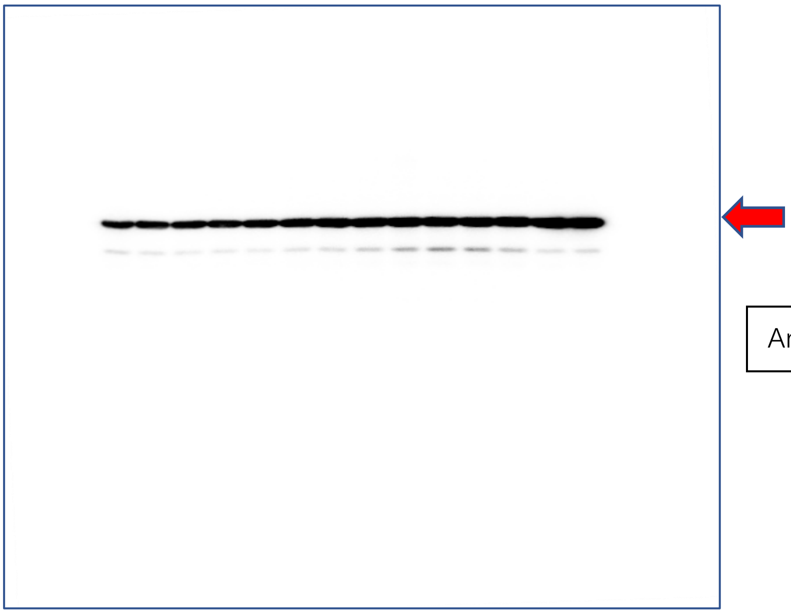

Anti-GAPDH

Figure 2B original picture (PROLIFERATIVE VS SECRETORYn=12 vs 12)

Endometrium mid-secretory phase (n=6) vs proliferative phase (n=6)

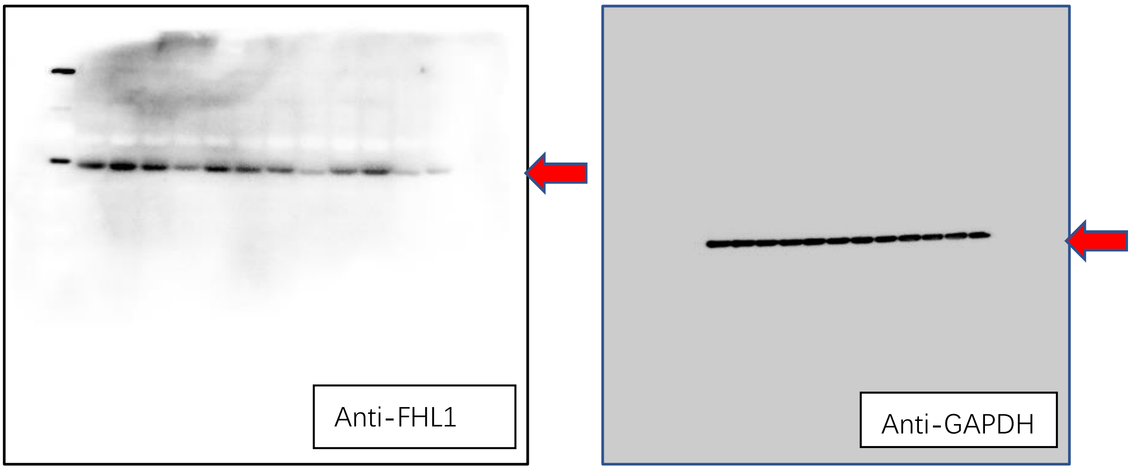

Endometrium mid-secretory phase (n=6) vs proliferative phase (n=6)

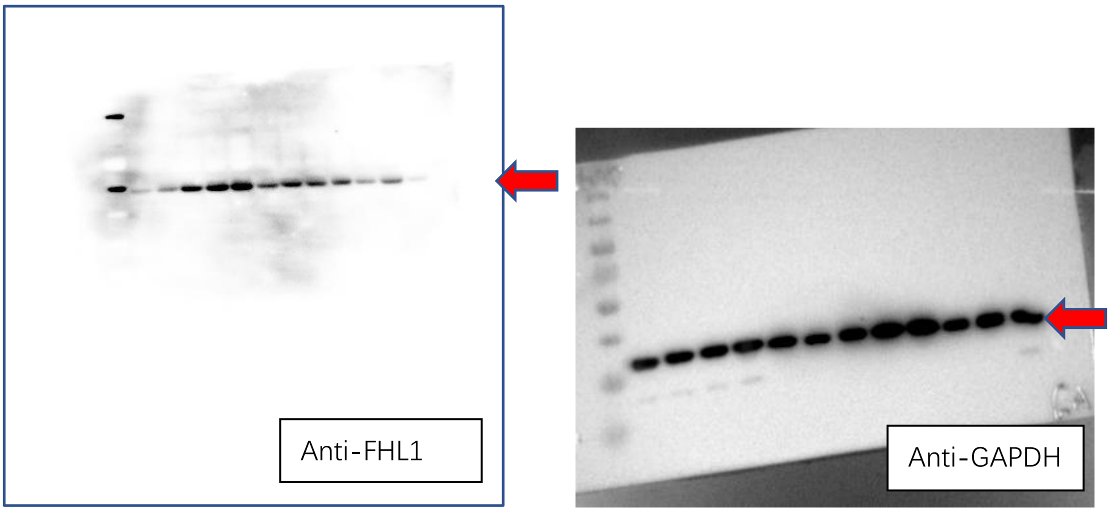

Figure 2F original picture

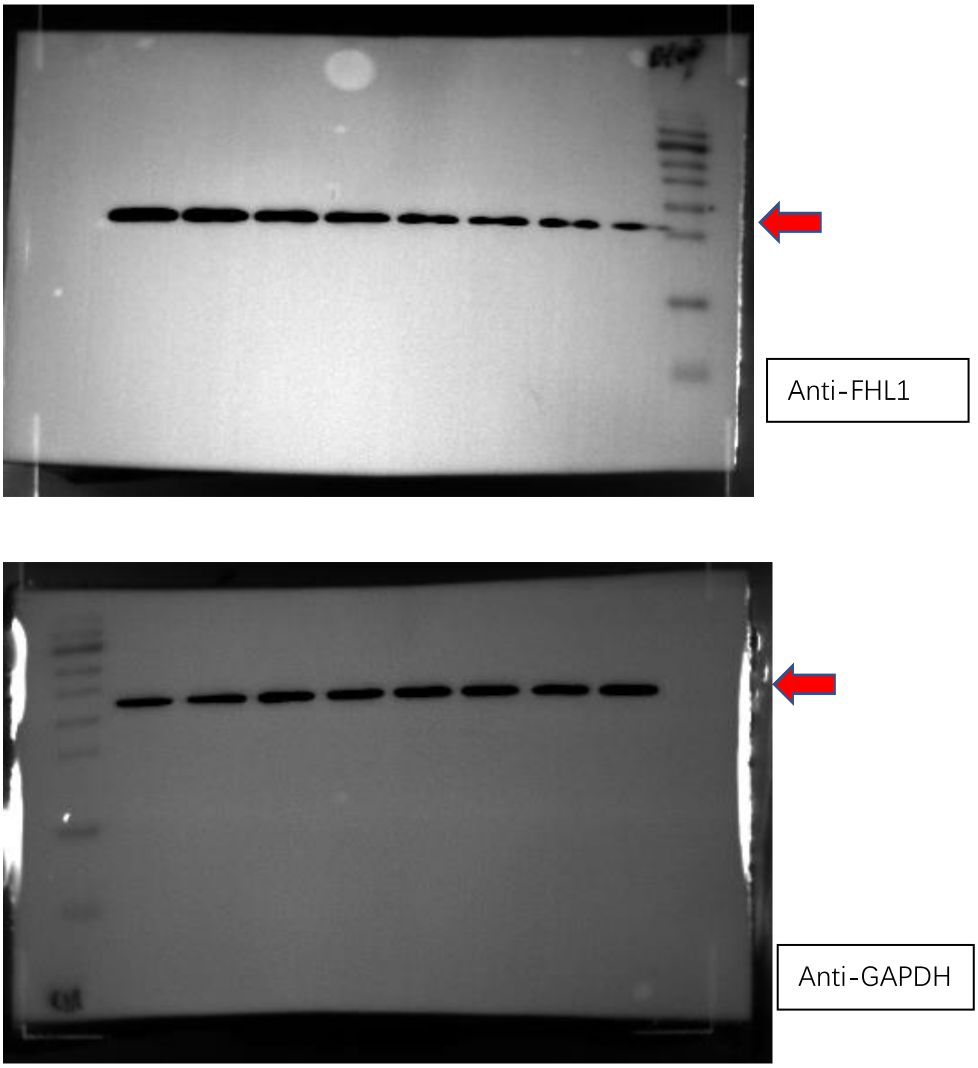

Figure 3C   original picture

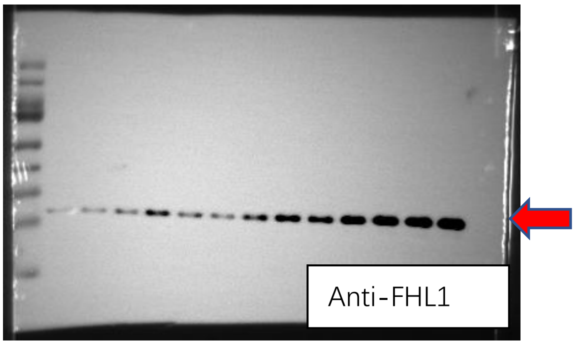

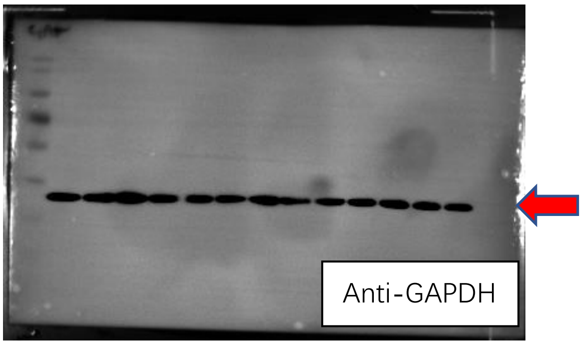

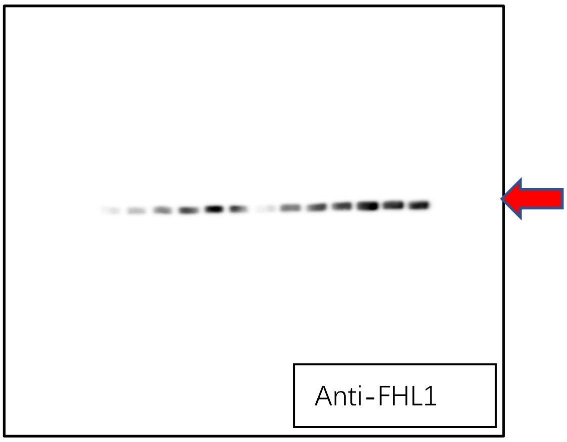

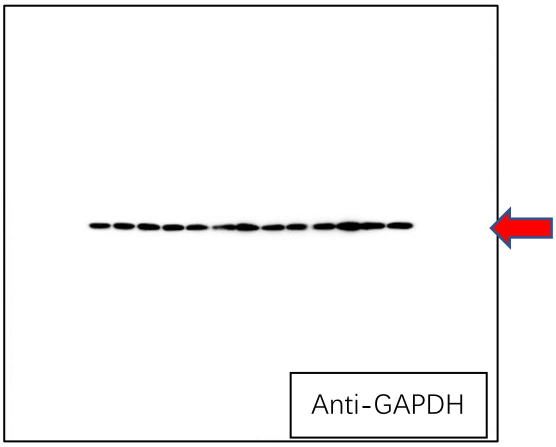

Figure 4B   original picture

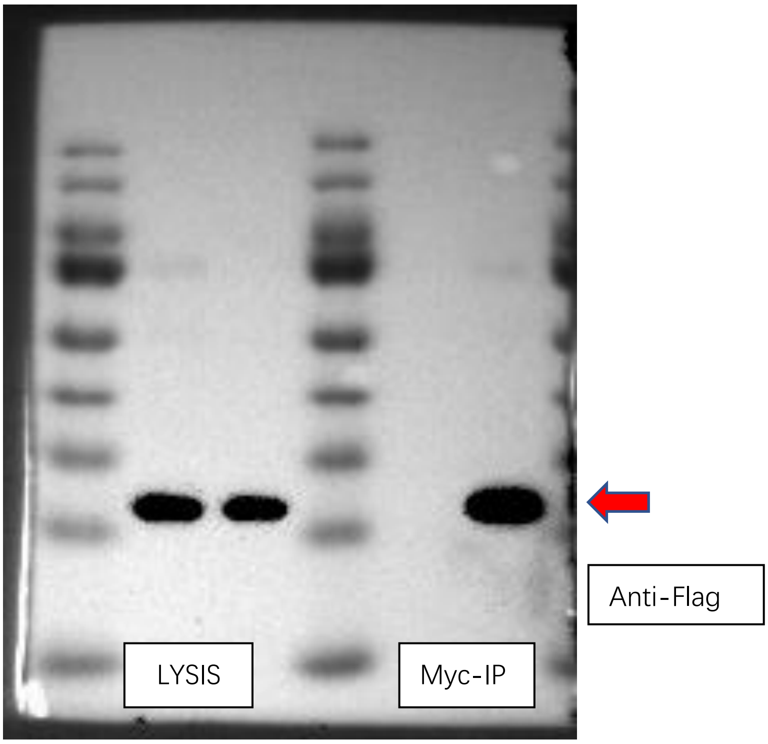

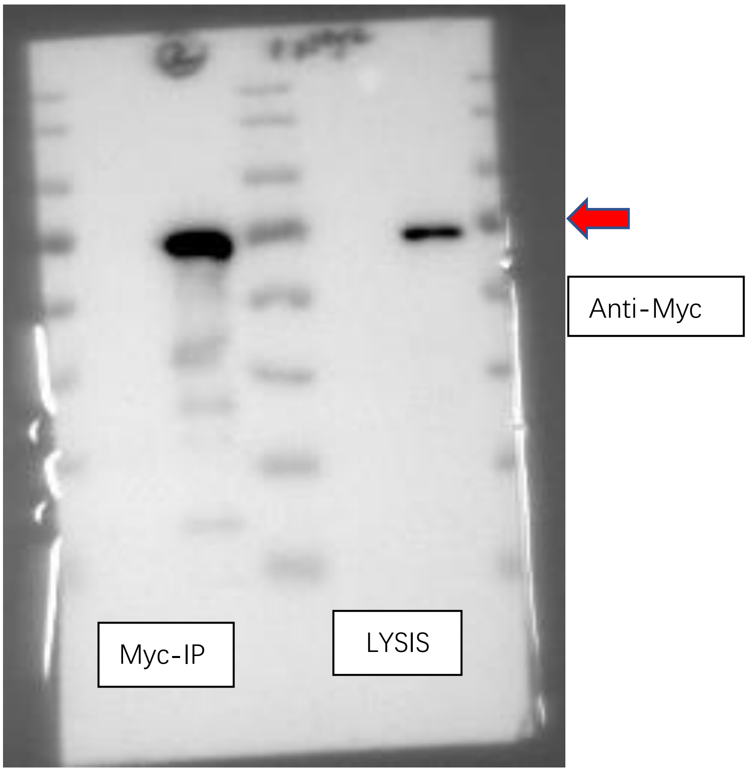

Figure 4C original picture

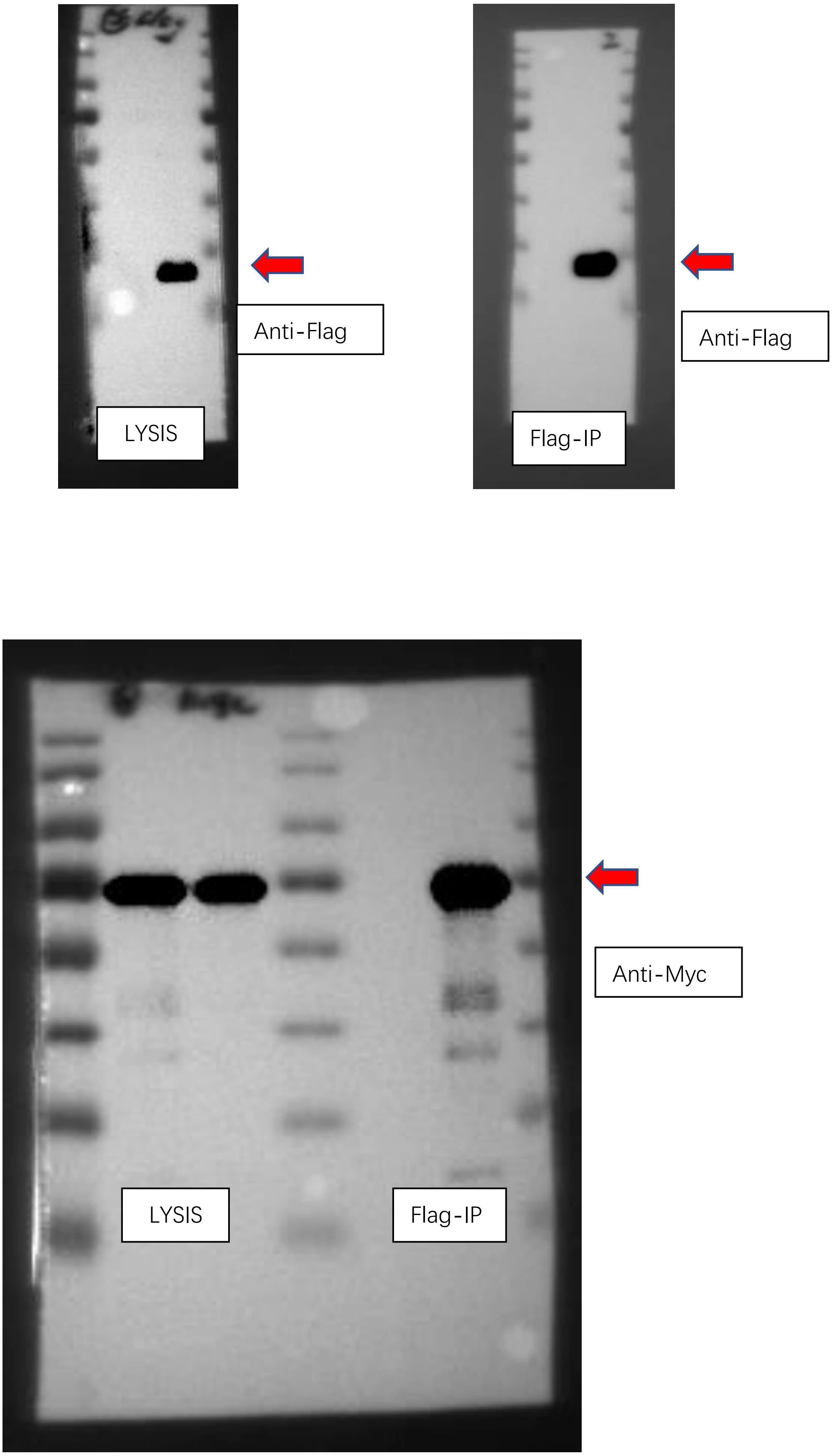

Figure 4D original picture

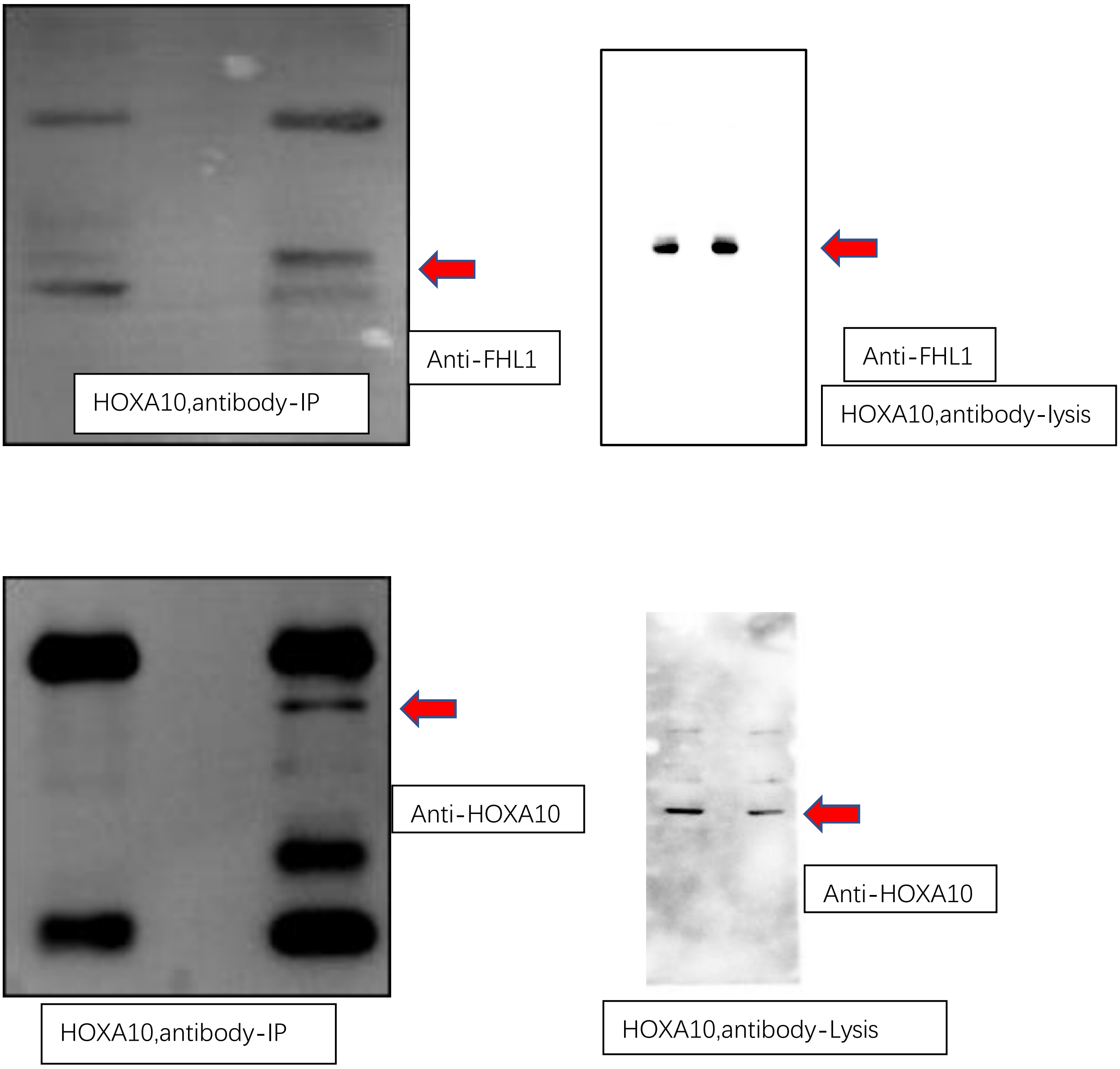

Figure 4F original picture

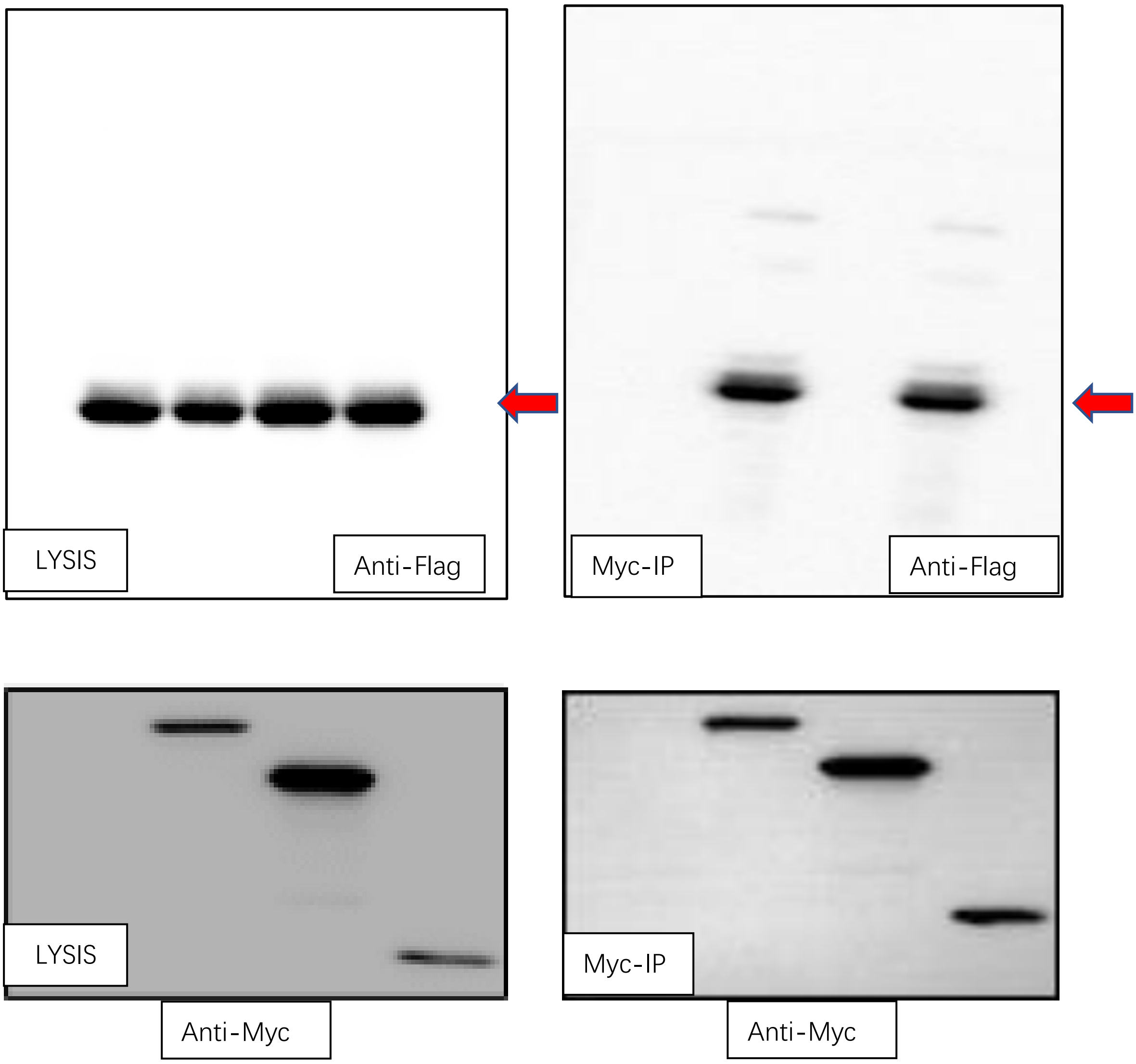

Figure 5B original picture

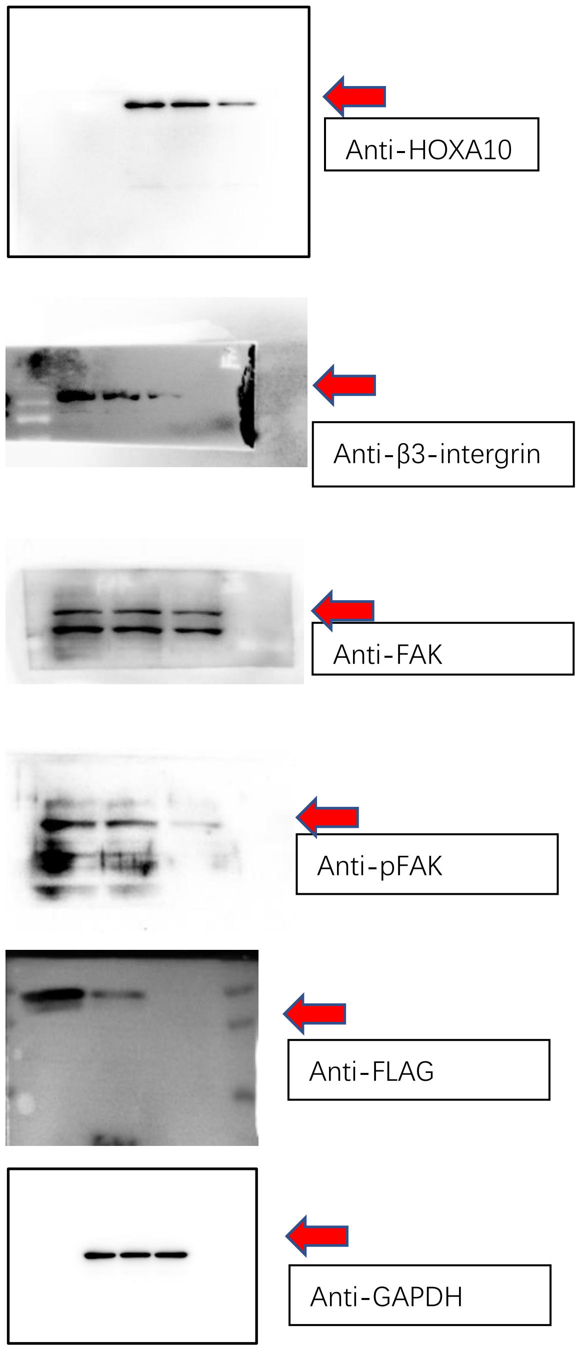

Figure 5C original picture

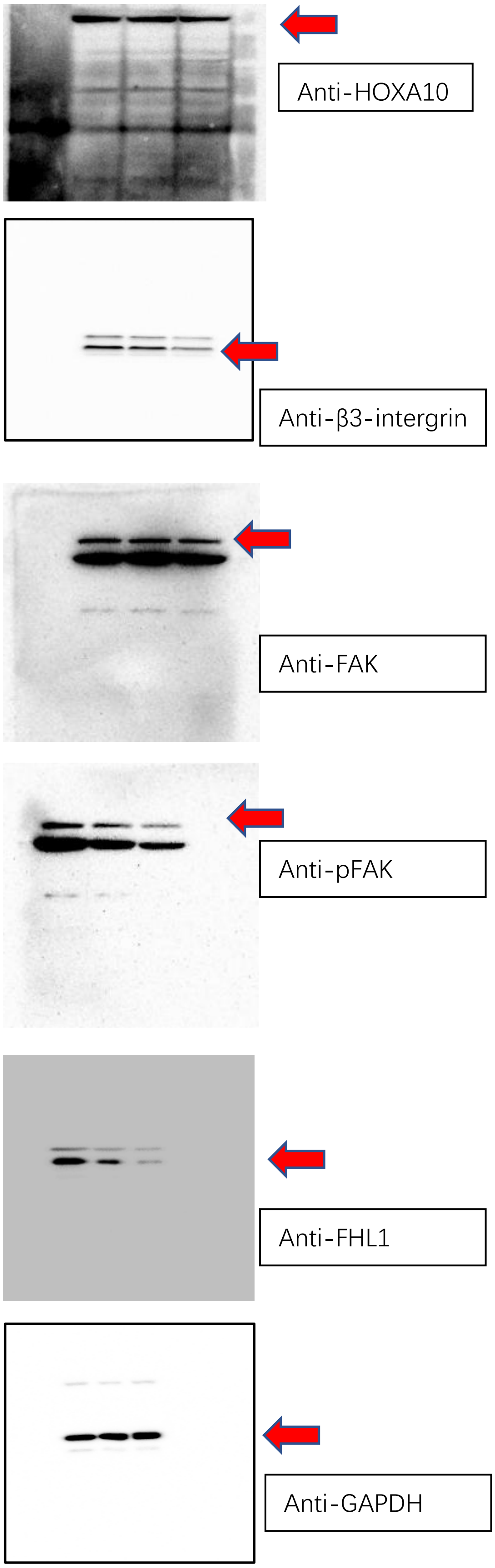

Figure 5E original picture

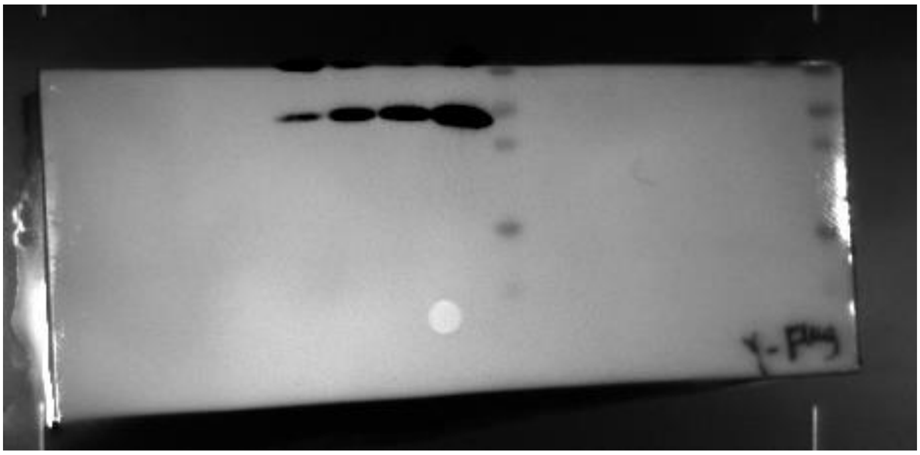

Anti-Flag

Anti-Myc

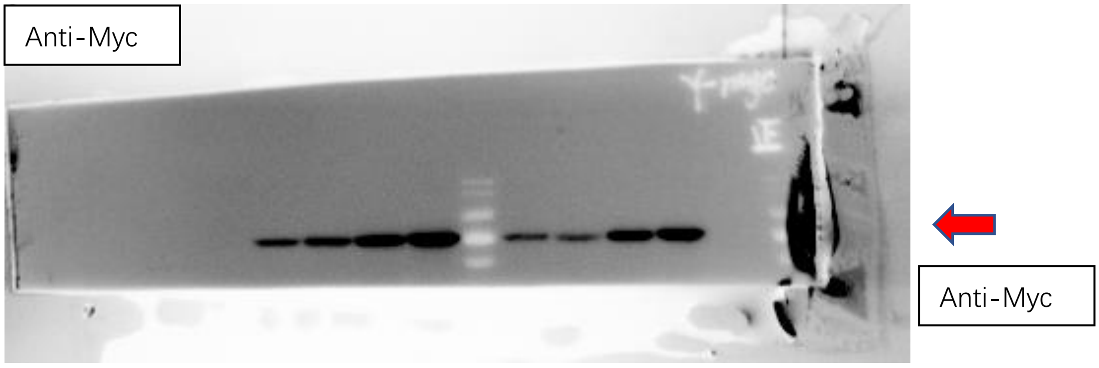

Anti-Myc

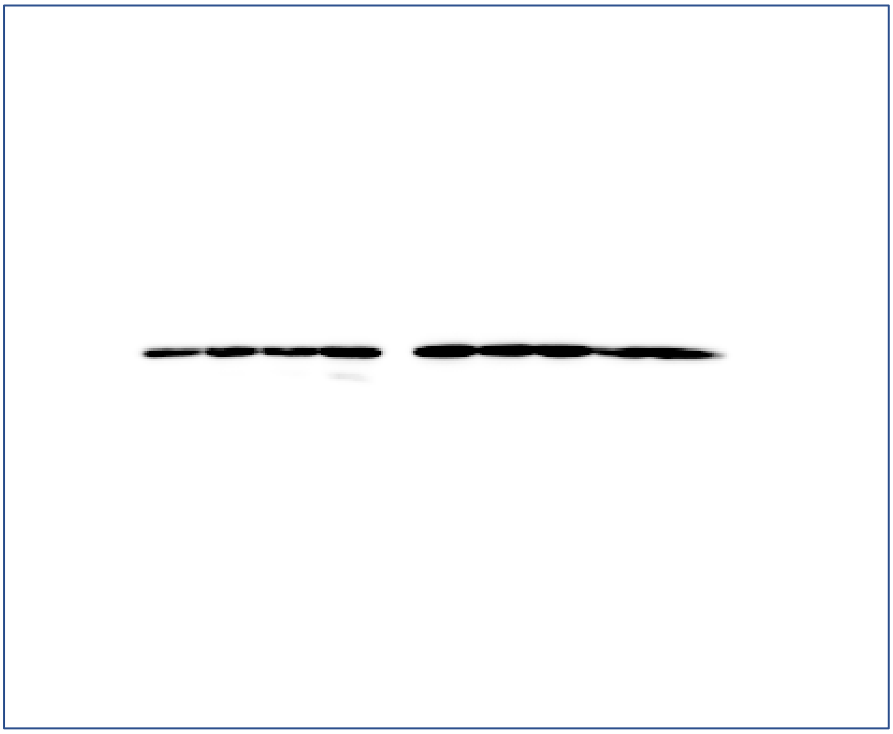

Anti-GAPDH

Figure 5F original picture

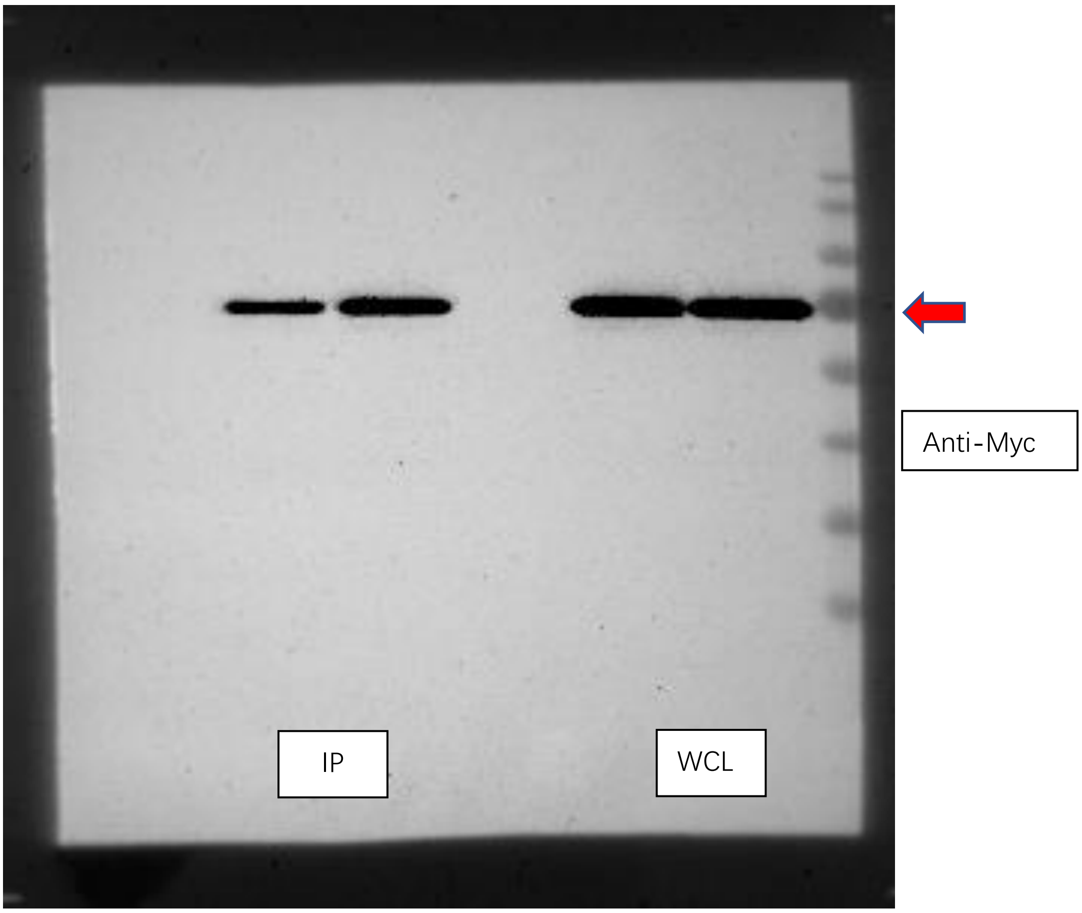

Anti-Myc

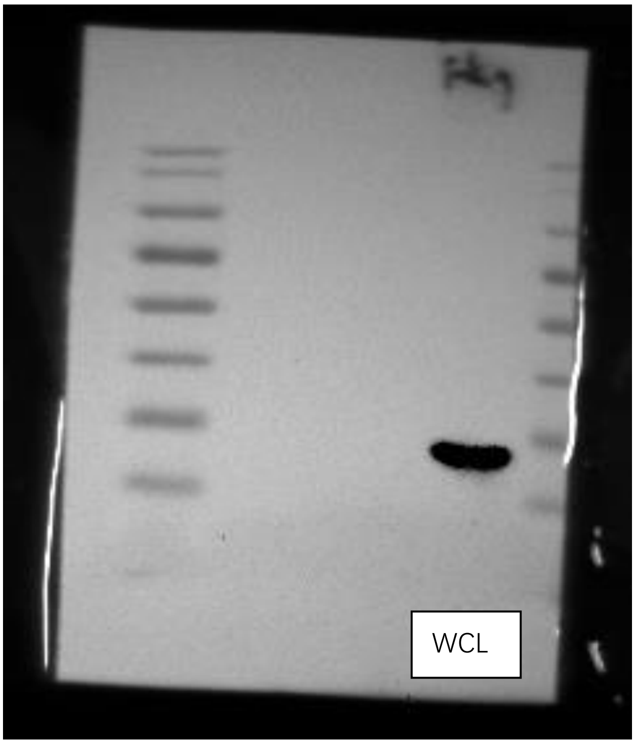

Anti-Flag

Figure 6C original picture

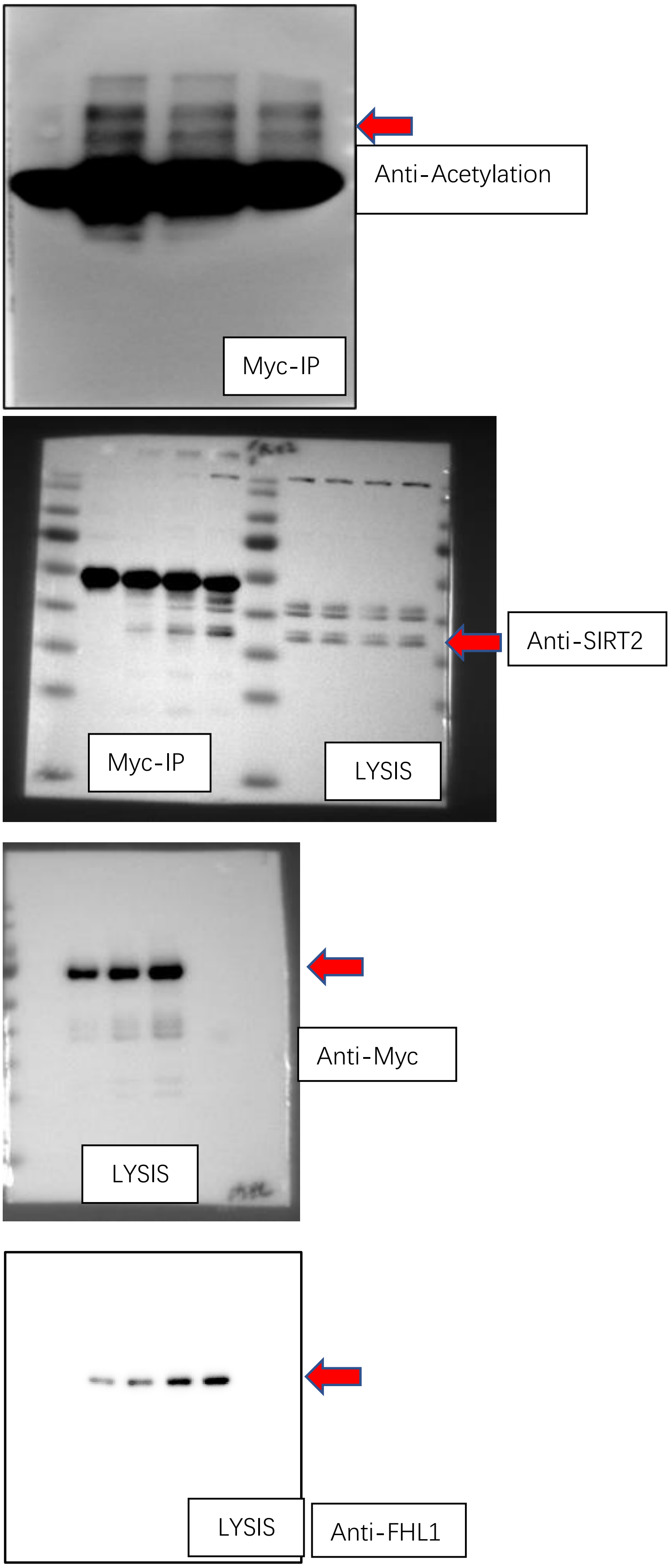

Figure 6D original picture

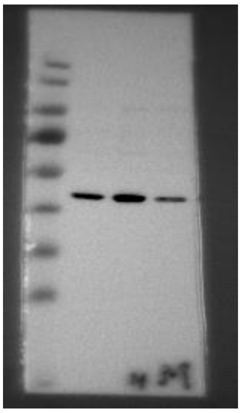

Anti-HOXA10

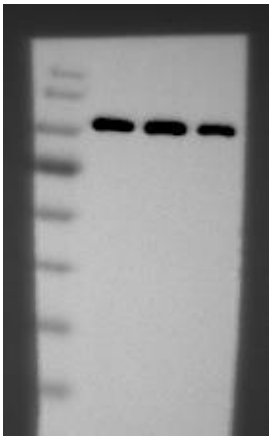

Anti-β3-intergrin

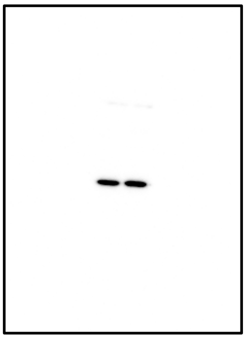

Anti-Flag

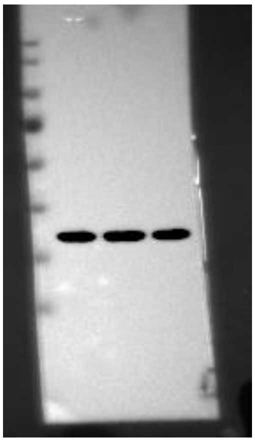

Anti-GAPDH

Figure 7B original picture

Endometrium FER(n=7) vs RIF(n=7)

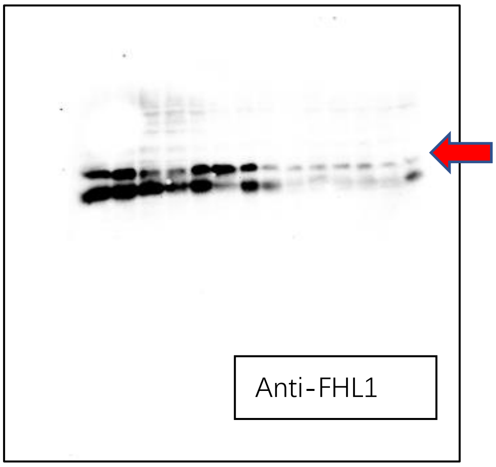

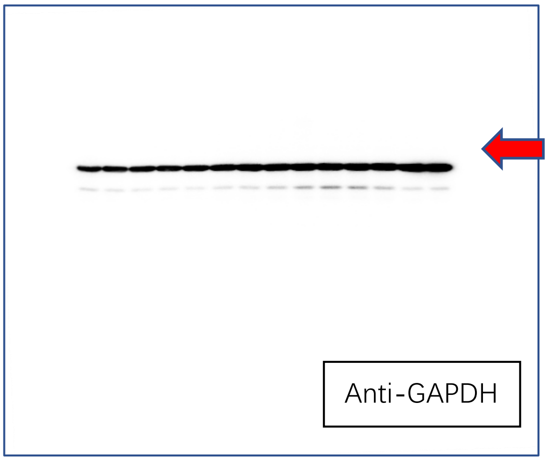

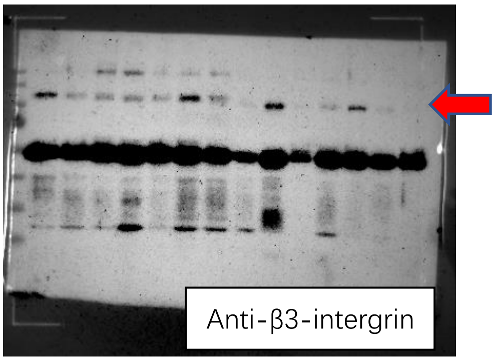

Endometrium FER(n=7) vs RIF(n=7)

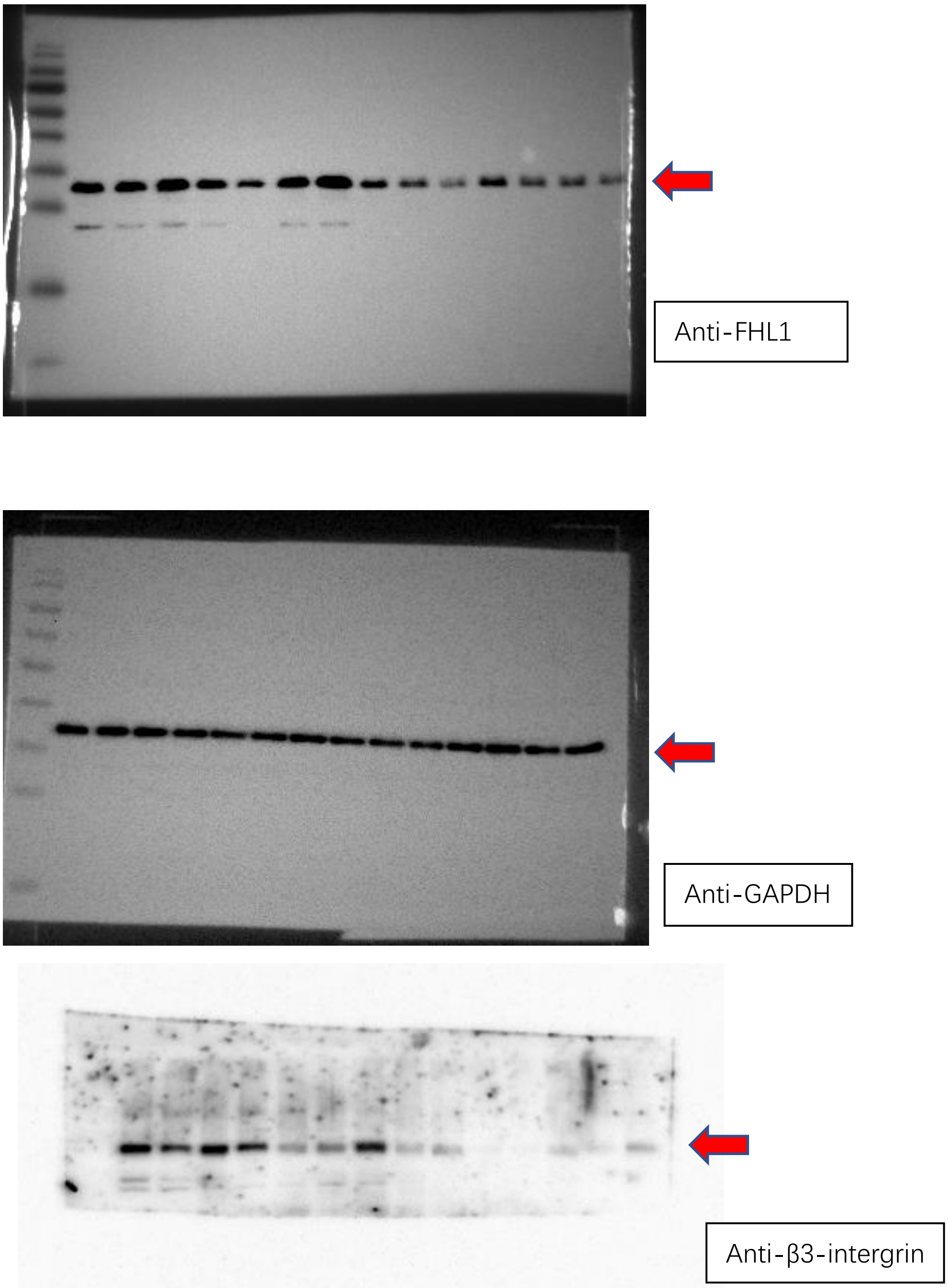

Supplement: Supplementary file 4 — Original Data File [file 41420_2022_1253_MOESM4_ESM.pdf]
